# Marine-Derived Indole Alkaloids and Their Biological and Pharmacological Activities †

**DOI:** 10.3390/md20010003

**Published:** 2021-12-21

**Authors:** Joko Tri Wibowo, Peni Ahmadi, Siti Irma Rahmawati, Asep Bayu, Masteria Yunovilsa Putra, Anake Kijjoa

**Affiliations:** 1 Research Center for Biotechnology, Life Sciences Research Organization, National Research and Innovation Agency (BRIN), Jl. Raya Bogor Km. 46, Cibinong 16911, Indonesia; joko.tri.wibowo@brin.go.id (J.T.W.); peni.ahmadi@brin.go.id (P.A.); siti.irma.rahmawati@brin.go.id (S.I.R.); asep044@brin.go.id (A.B.); 2 ICBAS-Instituto de Ciências Biomédicas Abel Salazar and CIIMAR, Universidade do Porto, Rua de Jorge Viterbo Ferreira, 228, 4050-313 Porto, Portugal

**Keywords:** indole alkaloids, marine natural products, bioactive compounds, marine-derived fungi, marine sponges

## Abstract

Novel secondary metabolites from marine macroorganisms and marine-derived microorganisms have been intensively investigated in the last few decades. Several classes of compounds, especially indole alkaloids, have been a target for evaluating biological and pharmacological activities. As one of the most promising classes of compounds, indole alkaloids possess not only intriguing structural features but also a wide range of biological/pharmacological activities including antimicrobial, anti-inflammatory, anticancer, antidiabetic, and antiparasitic activities. This review reports the indole alkaloids isolated during the period of 2016–2021 and their relevant biological/pharmacological activities. The marine-derived indole alkaloids reported from 2016 to 2021 were collected from various scientific databases. A total of 186 indole alkaloids from various marine organisms including fungi, bacteria, sponges, bryozoans, mangroves, and algae, are described. Despite the described bioactivities, further evaluation including their mechanisms of action and biological targets is needed to determine which of these indole alkaloids are worth studying to obtain lead compounds for the development of new drugs.

## 1. Introduction

Marine natural products (MNPs) have drawn much attention from both natural product chemists and pharmacologists due to their potential bioactivities. These chemical compounds have made a major contribution for the treatment of a myriad of diseases including cancer and infectious diseases as well as other therapeutic areas such as multidrug resistances (MDRs), cardiovascular diseases, and multiple sclerosis [1,2]. Compared to conventional synthetic molecules, MNPs present several advantages due to their unique structural features and complex scaffolds that are beneficial for drug discovery. MNPs usually possess high molecular weight and a large number of carbon and oxygen atoms. Moreover, marine-derived bioactive compounds have fewer but unique nitrogen and halogen atoms.

**Figure 1:**
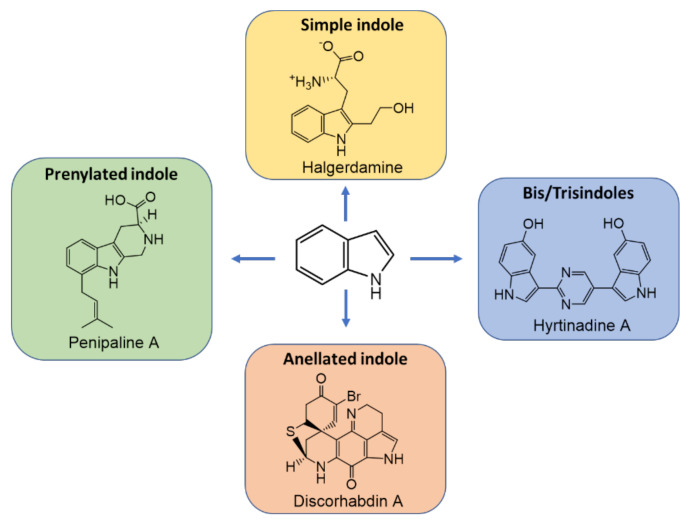
Classification of indole alkaloids based on chemical structures.

Alkaloids are a class of compound that contain organic nitrogenous bases [3]. Marine-derived alkaloids, especially indole alkaloids, exhibit a myriad of biological activities such as antibacterial, antifungal, anti-inflammatory, antileishmanial, antiplasmodial, anti-HIV, cytotoxic, glucose uptake stimulatory, larvicidal, trypanocidal, vasodilatory, and anti-cholinesterase activities [4]. Moreover, the alkaloids obtained from marine organisms frequently possess novel scaffolds that are not found in terrestrially related organisms [3]. Indole alkaloids are a class of alkaloids containing an indole moiety. Generally, indole alkaloids can be classified into four groups, *viz.* simple indole alkaloids, prenylated indoles, bis-/tris-indoles, and annelated indoles [5] (Figure 1). Simple indole alkaloids are mostly derived from the amino acid tryptophan (Trp) or its direct precursor, indole, in microorganisms or plants. On the other hand, prenylated indoles represent diverse groups of indole alkaloids that are commonly derived from Trp and isoprene unit(s) whereas bis- and tris-indoles are biosynthetically derived from two or three indole building blocks. For annelated indoles, a single indole core is fused with non-prenyl-derived (hetero)cyclic ring systems.

Besides several specific reviews on marine-derived indole alkaloids focusing on their biological and pharmacological activities [3,4,6], a comprehensive review by Netz and Opatz [5] has previously been published. In the present review, we have updated the indole alkaloids, isolated from various marine macro- and microorganisms including fungi, bacteria, sponges, bryozoans, mangrove plants, and algae during the period of January 2016 to October 2021. However, we only report the previously undescribed compounds isolated during this period, while the compounds reported before this period and are mentioned in the references used in this review are not included. The relevant biological and pharmacological activities of some potential alkaloids are also highlighted. The search engines used to find the compounds in this review were PubMed, MEDLINE, Web of Science, and Scopus.

Since our main research topics involved a discovery of new secondary metabolites from marine-derived fungi, marine invertebrates, and algae, which led to the isolation of a number of undescribed and structurally diverse indoles such as prenylated and annelated indoles with antibacterial and antibiofilm activities, this topic can provide us and other researchers who work with marine natural products to gain more insight into this class of compounds in both structural and biological activity aspects. 

## 2. Sources of Marine-Derived Indole Alkaloids Isolated from January 2016 to October 2021

**Figure 2:**
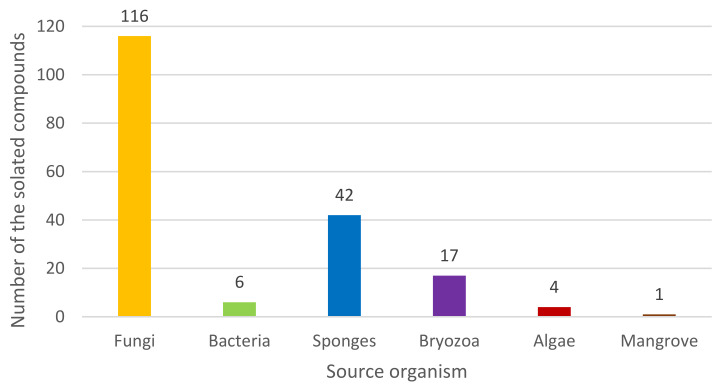
Distribution of the marine-derived indole alkaloids isolated from source organisms from January 2016 to October 2021.

The marine ecosystem has raised the awareness of natural product chemists due to the prospects of biologically active MNPs as a great potential for pharmaceuticals and cosmeceuticals. One of the most interesting classes of MNPs is indole alkaloids. Based on the literature search, 186 previously undescribed marine indole alkaloids that were isolated from various phyla of marine organisms including fungi, bacteria, sponges, bryozoans, mangrove trees, and algae have been reported between January 2016 and October 2021 (Figure 2). Intriguingly, many of these indole alkaloids possess unique structural features as well as interesting biological and pharmacological activities. 

### 2.1. Marine Microorganisms 

#### 2.1.1. Marine-Derived Fungi

Marine-derived fungi have been shown to be a prolific source of indole alkaloids, especially the prenylated indole alkaloids. For practical aspects and a better insight for the readers, the previously unreported indole alkaloids isolated from marine-derived fungi (and other marine organisms) are divided into four groups as above-mentioned. 

##### Simple Indole Alkaloids

Although marine sponges and soft coral-associated fungi are rich sources of indole alkaloids, other marine invertebrates such as sea star and sea cucumber are also found to host indole alkaloid-producing fungi. In order to facilitate the perception, the compounds of this group are not ordered according to the type of host organisms, but according to the complexity of the substituents of the indole core, starting from acyclic substituents to cyclic ones. For cyclic substituents, the order of the compounds is from monocyclic and progressing to bi-, tri-, and polycyclic, respectively.

**Figure 3:**
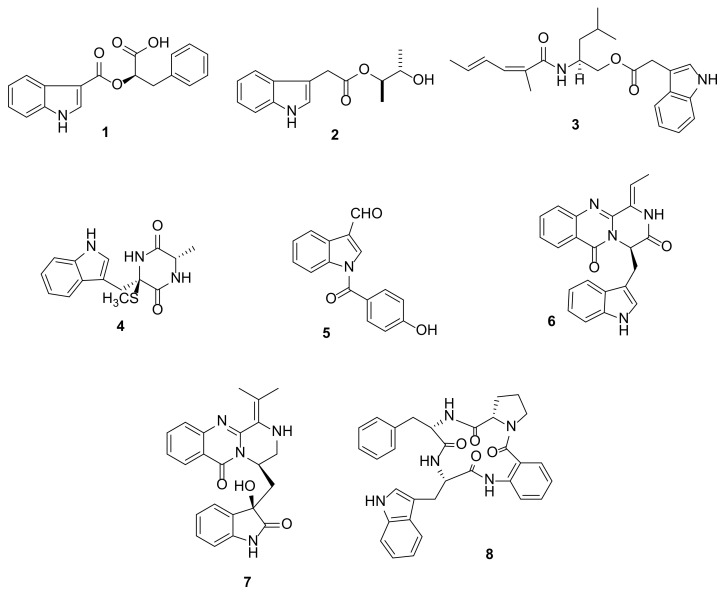
Structures of **1**–**8**.

Supplementation of L-Trp into Glucose-Yeast-Peptone (GYP) culture medium of the fungus *Fusarium* sp. L1, which was isolated from the inner tissue of a sea star *Acanthaster planci* collected from the Xisha Islands in China, resulted in the production of two previously unreported simple indole alkaloids, fusariumindole C (**1**) and (±)-isoalternatine A (**2**) (Figure 3) [7]. 

A marine-derived fungus *Dichotomomyces cejpii* F31-1, obtained from the inner tissue of a soft coral *Lobophytum crassum* that was collected from the Hainan Sanya National Coral Reef Reserve, China, yielded an indole-containing amide, dichotomocej D (**3**) (Figure 3) [8]. Pseudellone D (**4**) (Figure 3), a metylsulfanyldiketopiperazine-containing indole, was also obtained from the culture of the fungus *Pseudallescheria ellipsoidea* F42-3, which was isolated from the inner tissue of the soft coral *L. crassum* collected from the Hainan Sanya National Coral Reef Reserve, China [9]. 

A marine fungus *Engyodontium album* (IVB1b), isolated from a marine sponge *Ircinia variabilis* that was collected at İlyosta–Ayvalık in Balıkesir, Turkey, produced 1-(4-hydroxybenzoyl)indole-3-carbaldehyde (**5**) (Figure 3) [10]. 

Mycelia of a fungus *Scedosporium apiospermum* F41-1, also obtained from the inner tissue of the soft coral *L. crassum* that was collected from the Hainan Sanya National Coral Reef Reserve, China, yielded scequinadoline J (**6**) (Figure 3). The absolute configuration of the stereogenic carbon (C-14) in **6** was established by a comparison of the calculated and experimental electronic circular dichroism (ECD) spectra [11]. Interestingly, the same fungus produced different metabolites including an undescribed pyrazinoquinazolinone-containing indole, scequinadoline G (**7**), when cultured in the GYP medium supplementation of L-Trp, L-Phe, L-Thr, and D,L-Met from when it was cultured solely in GPY medium [12]. 

Sartoryglabramide B (**8**) (Figure 3), an indole-containing tetrapeptide, was isolated from the culture of a marine sponge-associated fungus *Neosartorya glabra* KUFA 0702A, which was isolated from the marine sponge *Mycale* sp., collected at Samaesarn Island in the Gulf of Thailand. The absolute configurations of the amino acid constituents were determined by X-ray analysis and confirmed by chiral high performance liquid chromatography (HPLC) analysis of its hydrolysate and compared with D- and L-amino acid standards [13]. 

##### Prenylated Indoles

Similar to the simple indole alkaloids, the prenylated indoles are not ordered according to the producing fungi, but according to their structural complexity, starting from a simple indole core with acyclic prenyl substituents to cyclic prenyl substituents, progressing to the indole core fused with a prenyl-derived ring system, and the number of rings fused with the indole core determines their degree of complexity. 

**Figure 4:**
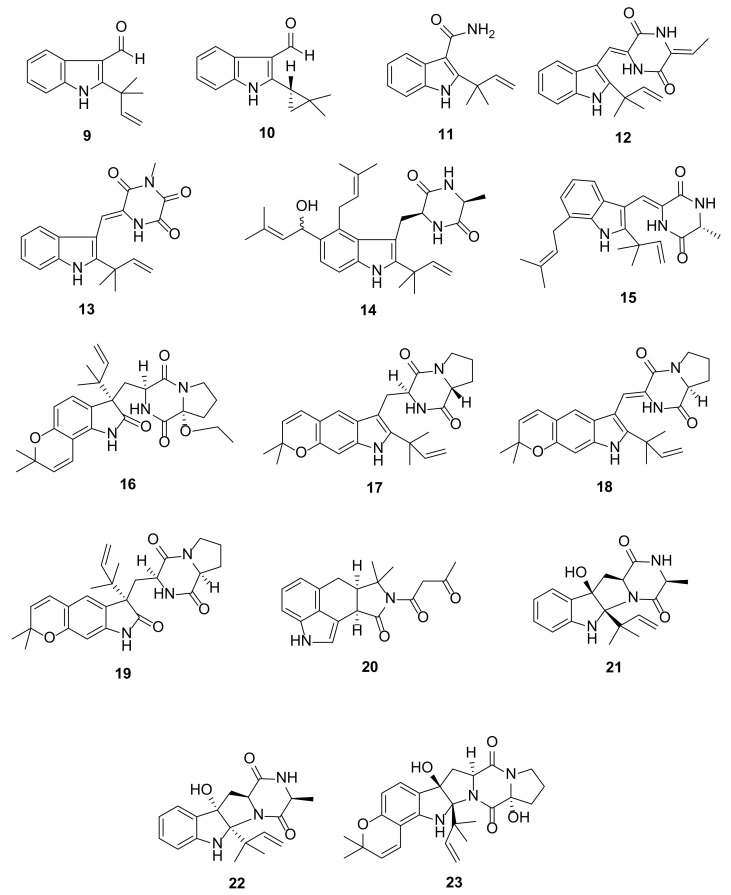
Structures of **9**–**23**.

The extract of a solid rice culture of a mangrove-derived fungus *Eurotium chevalieri* KUFA 0006, isolated from a healthy twig of *Rhizophora mucronata* Poir., which was collected at Kung Krabaen Bay Royal Development Study Center, yielded two prenylated indoles, 3-carbaldehyde derivatives, namely, 2-(2-methyl-3-en-2-yl)-1*H*-indole-3-carbaldehyde (**9**) and 2-(2,2-dimethylcyclopropyl)-1*H*-indole-3-carbaldehyde (**10**) (Figure 4) [14], whereas an amide-containing eurotiumin D (**11**) and a diketopiperazine-containing eurotiumin C (**12**) (Figure 4) were obtained from the extract of a liquid culture of *Eurotium* sp. SCSIO F452, which was isolated from a South China Sea sediment sample [15]. 

The diketopiperazine-containing indole alkaloids 11-methylneoechinulin E (**13**), variecolorin M (**14**), and (+)-variecolorin G (**15**) (Figure 4) were reported from a culture extract of *Aspergillus* sp. EGF 15-0-3 isolated from an unidentified soft coral that was collected from the South China Sea [16]. Co-cultivation of marine sediment-derived fungi *Aspergillus sulphureus* KMM 4640 and *Isaria felina* KMM 4639, on a solid rice medium, resulted in the isolation of a diketopiperazine-containing prenylated indole, 17-*O*-ethylnotoamide M (**16**) (Figure 4) belonging to a notoamide family [17]. The solid rice culture extract of *Aspergillus versicolor*, which was collected from the mud in the South China Sea, produced several prenylated indole alkaloids including the previously unreported asperversamides F-H (**17**–**19**) (Figure 4) featuring a pyrano[3,2-*f*]-indole. The structures of **17**–**19** were elucidated by extensive 1D and 2D NMR spectral analysis. Compound **17** is a C-17 epimer of the previously described dihydrocarneamide A, whose absolute structure was determined by a single-crystal X-ray diffraction analysis [18]. The absolute configurations of C-11 and C-17 in **17** were confirmed as 11*S*, 17*R* by comparison of the calculated and experimental optical rotations. On the other hand, the absolute configuration of the stereogenic carbon (C-17) in **18** was determined by comparison of the calculated and experimental ECD spectra while the absolute configurations of C-3, C-11, and C-11 in **19** were established by X-ray diffraction analysis using CuKα radiation [18]. 

The ethyl acetate (EtOAc) extract of the culture of *Pseudallescheria boydii* F19-1, which was isolated from the inner tissue of a soft coral *L. crassum*, collected in the Hainan Sanya National Coral Reef Reserve, China, yielded a cyclopiazonic acid analogue, pseuboydone E (**20**) (Figure 4). The absolute configurations of C-4 and C-8 in **20** were established by comparison of the calculated and experimental ECD spectra [19]. 

The fungus *Eurotium* sp. SCSIO F452 also yielded tetracyclic 2,5-diketopiperazine-containg prenylated indoles eurotiumins A (**21**) and B (**22**) (Figure 4). Both **21** and **22** feature a hexahydropyrrolo[2,3-*b*]indole skeleton (derived from a linkage between C-2 of the indole moiety and a nitrogen atom of a 1,4-diketopiperazine) and occurred as a pair of diastereomers, differing only in the absolute configurations of C-2 and C-3. The absolute configurations of the stereogenic carbons in **21** and **22** were established as 2*S*, 3*R*, 9*S*, 12*S* for **21** and 2*R*, 3*S*, 9*S*, 12*S* for **22** by comparison of the calculated and experimental ECD spectra [15].

Co-culture of *A. sulphureus* KMM 4640 with *I. felina* KMM 4639 also yielded 17-hydroxynotoamide D (**23**) (Figure 4), consisting of a hexahydropyrrolo[2,3-*b*]indole skeleton, similar to that of **21** and **22**, which is angularly fused with a 2,2-dimethylpyran ring. The absolute configurations of the stereogenic carbons in **23** were established by comparison of the calculated and experimental ECD spectra [17].

**Figure 5:**
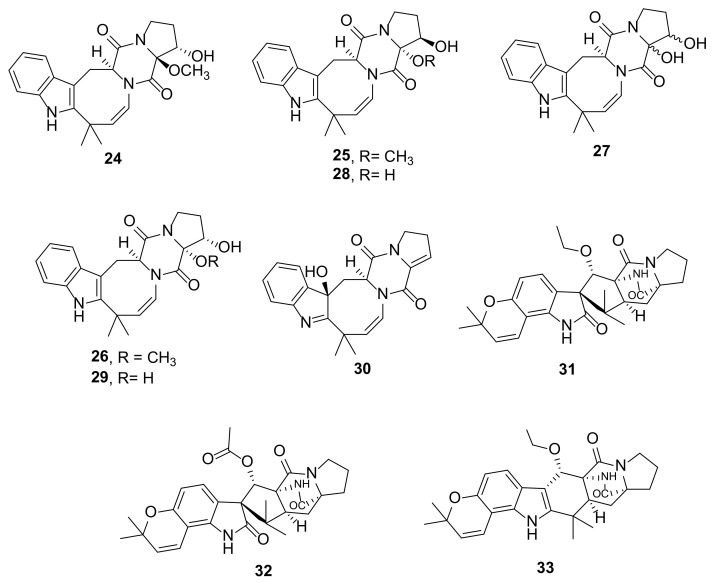
Structures of **24**–**33**.

The EtOAc extract of a solid rice culture of the fungus *Penicillium dimorphosporum* KMM 4689, isolated from soft coral samples that were collected from various points in the South China Sea, yielded seven unreported deoxyisoaustamide derivatives, namely 16α-hydroxy-17β-methoxydeoxydihydroisoaustamide (**24**), 16β-hydroxy-17α-methoxydeoxydihydroisoaustamide (**25**), 16α-hydroxy-17α-methoxydeoxydihydroisoaustamide (**26**), 16,17-dihydroxydeoxydihydroisoaustamide (**27**), 16β,17α-dihydroxydeoxydihydroisoaustamide (**28**), 16α,17α-dihydroxydeoxydihydroisoaustamide (**29**), and 3β-hydroxydeoxyisoaustamide (**30**) (Figure 5). The structures of the compounds of this group are characterized by a 6/5/8/6/5 pentacyclic ring system whose indole and 1,4-diketopiperazine moieties are linked through the eight-membered hexahydroazocine ring. The structures of **24**–**30** were elucidated by extensive analysis of their 1D and 2D NMR spectra. The relative configurations of the stereogenic carbons in **24** were determined by single-crystal X-ray analysis whereas those of **25**–**30** were established by NOESY correlations. The absolute structure of **24** was based on its biogenic relationship with that of the previously described (+)-deoxyisoaustramide. The absolute configurations of the stereogenic carbons of the rest of the compounds were based on their biogenetic consideration as well as a comparison of their CD spectra [20].

The co-culture of the marine-derived *A. sulphureus* KMM 4640 with *I. felina* KMM 4639 also produced the unreported notoamides including 10-*O*-ethylsclerotiamide (**31**), 10-*O*-acetylsclerotiamide (**32**), and 10-*O*-ethylnotoamide R (**33**) (Figure 5) [17].

**Figure 6:**
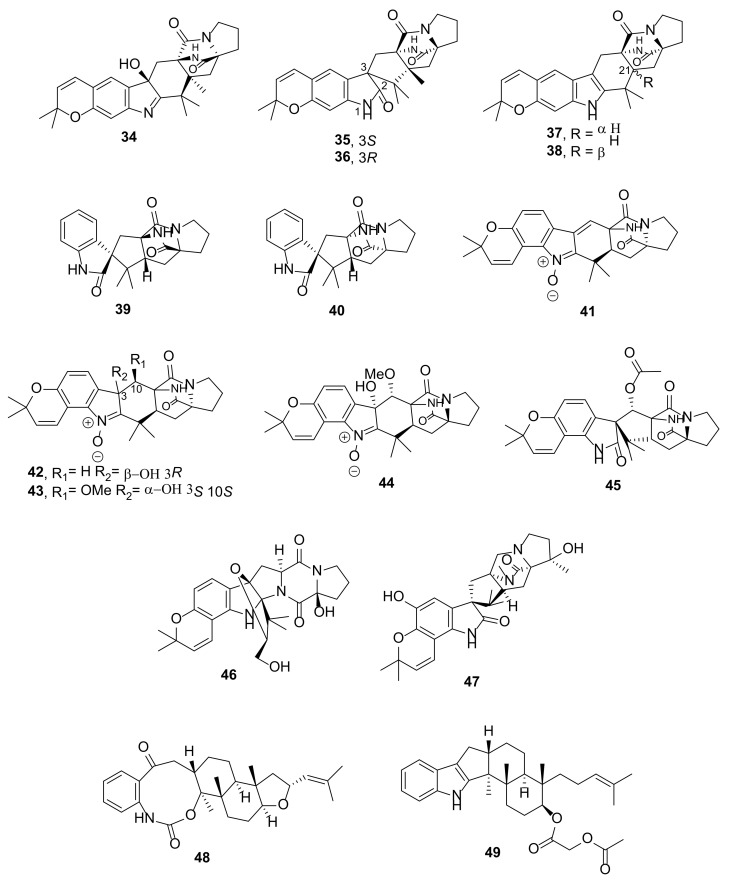
Structures of **34**–**49**.

*Aspergillus versicolor* from the mud, collected in the South China Sea, also produced rare linearly fused dimethypyranoindoles containing an amine fused-imine pyrrole ring system, asperversamides A–E (**34**–**38**) (Figure 6). Compounds **34**–**36** and **38** each contain a rare *anti* bicyclo[2.2.2]diaza-octane ring, while **37** contains an analog *syn* ring. Moreover, **35**/**36** and **37**/**38** are pairs of C-3 and C-21 epimers, respectively. The relative configurations of all the compounds were established by NOESY correlations. In the case of **34**, the absolute configurations of its stereogenic carbons were established by comparison of the calculated and experimental ECD spectra, while the absolute structure of **35** was established by the characteristic positive Cotton effect (CE) at 223 nm (+25.9) and negative CE at 242 nm (−28.6) in the experimental ECD spectrum and confirmed by a single-crystal X-ray diffraction analysis. The absolute configurations of the stereogenic carbons in **37** and **38** were established based on a similarity of their ECD spectra to that of the previously reported compound, (+)-6-*epi-*stephacidin A, and in the case of **37**, its absolute structure was confirmed by X-ray analysis [18].

Brevianamides X (**39**) and Y (**40**) (Figure 6), two spiro 2-oxindole containing a bicyclo[2.2.2]diaza-octane ring system, were obtained from an acetone extract of the culture of a marine-derived fungus *Penicillium brevicompactum* DFFSCS025, which was isolated from a deep-sea sediment sample collected in the South China Sea in Hainan Province, China. The planar structures of both compounds were established by detailed analysis of 1D and 2D NMR spectra. NOESY correlations were used to determine the relative configurations of the stereogenic carbons of both compounds while their absolute configurations were established by a comparison of the calculated and experimental ECD spectra. Interestingly, **39** was found to be a diastereomer of the previously described (−)-depyranoversicolamide B [21].

The ethyl acetate extract of a culture of *Aspergillus* sp. YJ191021, isolated from a soil sample collected from the intertidal zone of Zhoushan, Zhejiang, China, yielded unreported prenylated diketopiperazine indole alkaloids, asperthins A–F (**41**–**46**) (Figure 6). Compounds **41**–**44** share the same feature consisting of a bicyclo[2.2.2]diaza-octane-containing indole *N*-oxide, which is angularly fused with a dimethylpyran moiety, while **45** is a bicyclo[2.2.2]diaza-octane-containing spiro 2-oxindole, angularly fused with dimethyl pyran. Compound **46** features an angularly fused pyranoindole containing a diketopiperazine-fused amine pyrrole ring system. The planar structures of **41**–**46** were established by extensive analysis of 1D and 2D NMR spectra while the absolute configurations of their stereogenic carbons were determined by comparison of the calculated and experimental ECD spectra [22].

The EtOAc extract of a culture of *Penicillium janthinellum* HK1-6, which was isolated from a mangrove rhizosphere soil collected from the Dongzhaigang mangrove natural reserve in Hainan Island, yielded an unreported paraherquamide J (**47**) (Figure 6) whose structure consists of a prenylated indole featuring a spiro oxindole angularly fused with a dimethylpyran ring, which is connected with a bicyclo[2.2.2]diaza-octane-containing diketopiperazine-fused amine pyrrole ring system. The relative configurations of the stereogenic carbons in **47** were established by NOESY correlations while their absolute configurations were established by comparison of its ECD spectrum with that of mangrovamide A, a previously reported compound whose absolute structure was established by ECD data and X-ray analysis [23].

The EtOAc extract of a culture of *Fusarium* sp. L1 from a GPY liquid medium supplemented with L-Trp also produced fusaindoterpenes A (**48**) and B (**49**) (Figure 6). Compound **48** is a rearranged prenylated indole containing a 8,9-dihydro-1,3-oxazonine-2,6 (3*H*,7*H*)-dione ring, while **49** features an indole fused with a substituted dodecahydro-1*H*-cyclopenta[*a*]naphthalene ring system. The relative configurations of **48** and **49** were established by NOESY correlations. The absolute configurations of the estereogenic carbons of **48** were established by X-ray analysis, while those of **49** were established by a comparison of the calculated and experimental ECD spectra [7].

**Figure 7:**
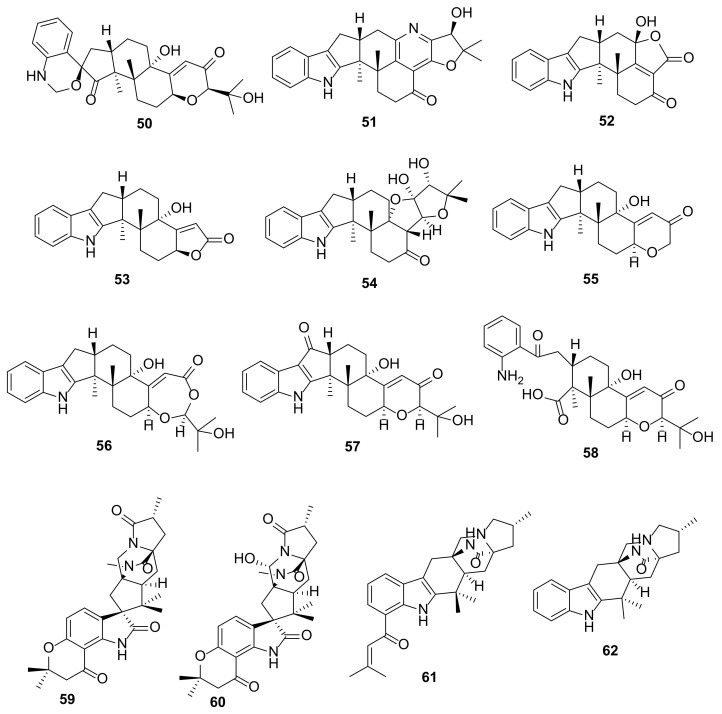
Structures of **50**–**62**.

The EtOAc extract of a fermentation broth of *Penicillium* sp. KFD28, isolated from a bivalve mollusc, *Meretrix lusoria*, which was collected from Haikou Bay, China, furnished four indolediterpenoids, named penerpenes A–D (**50**–**53**) (Figure 7). Compound **50** features a hexacyclic ring system comprising 1,2-dihydro-2′*H*-spiro [3,1-benzoxazine-4,1′-cyclopentan]-2′-one fused with a decalin ring system, while **51**–**53** share a common feature, having an indole ring system fused with a cyclopentane ring of the dodecahydro-1*H*-cyclopenta[a]naphthalene ring system. However, the structure of **51** is unique since it has a pyridine ring fused with a decalin moiety. The planar structures of **50**–**53** were elucidated by extensive analysis of their 1D and 2D NMR data. The absolute configurations of the stereogenic carbons in **50** and **51** were established by comparison of the calculated and experimental ECD spectra while only the relative configurations of **52** were established by ROESY correlations. On the other hand, the absolute configurations of the stereogenic carbons in **53** were established using the ECD exciton chirality models [24]. The presence of a spiro 1,4-dihydro-2*H*-3,1 benzoxazine ring in **50** and the presence of a pyridine ring in **51**, together with a new carbon skeleton in **52** and **53**, led Kong et al. [24] to propose the biosynthesis of these compounds as a degradation and rearrangement of the carbon skeletons of paxilline-type indole terpenoids, paxilline (I) and emindole SB (**VI**), which were co-isolated with **50**–**53**.

**Figure 8:**
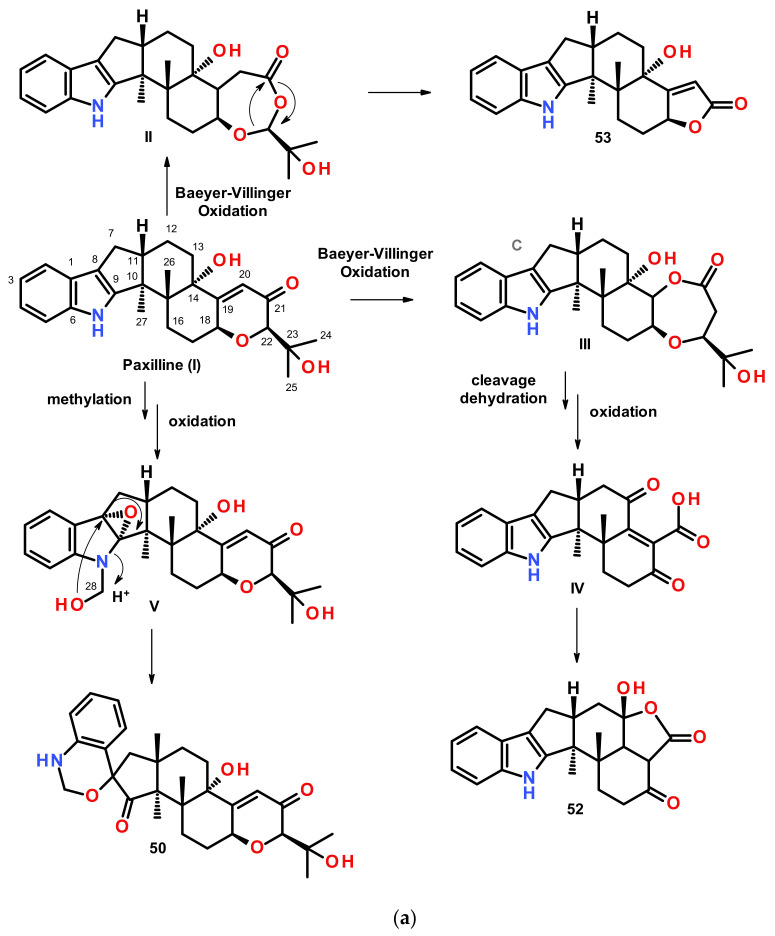

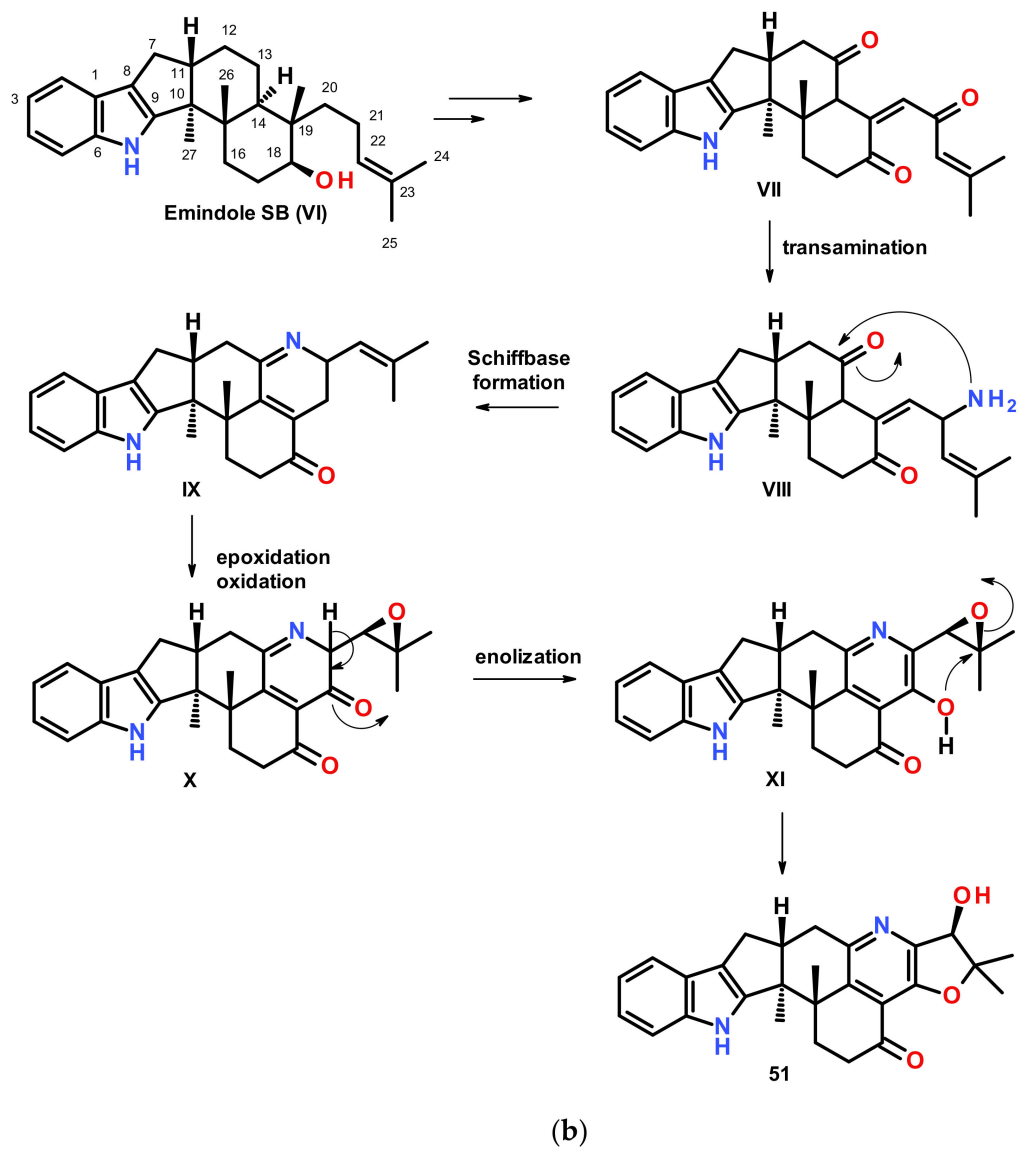
(**a**) Proposed biosynthetic pathways of **50**, **52**, and **53** from paxilline (**I**); (**b**) Proposed biosynthetic pathways to **51** from emindole SB (**VI**).

Compounds **50**, **52**, and **53** are hypothesized to derive from paxilline (**I**) (Figure 8a).

*N*-methylation and oxidation of the indole portion of paxilline (**I**) yields **V** with *N*-hydroxymethyl and an epoxide between C-8 and C-9. Nucleophillic substitution of C-8 by OH-28 with a concomitant opening of the epoxide ring and a cleavage of C-9-N leads to thee formation of a spiro 1,4-dihydro-2*H*-3,1 benzoxazine ring in **50.** On the other hand, Baeyer–Villinger oxidation of **I** gives **II**, with a 1,3-dioxepan-4-one ring**,** and **III**, with a 1,4-dioxepan-5-one ring. Rearrangement of the 1,3-dioxepan-4-one ring in **II** yields **53**, while ring cleavage, dehydration, and oxidation of **III** gives **IV**. Lactonization of **IV** yielded **52** (Figure 8a).

Emindole SB (**VI**) was proposed to be a biosynthetic precursor of **51**. Oxidation of **VI** gives rise to **VII**, followed by transamination to give **VIII**. Nucleophillic addition of the ketone at C-13 by NH_2_-21 yields an imine in **IX**. Oxidation and epoxidation of **IX** gives **X**, which, after enolization, yields **XI**. Nucleophillic substitution on C-23 of the epoxide by OH-18 leads to a formation of a 3-hydroxydihydropyran ring in **51** (Figure 8b).

Further investigation of the same fungal extract by the same research group led to the isolation of an additional five paxilline-type indolediterpenoids, penerpenes E–I (**54**–**58**) (Figure 7) [25]. Compound **54** has a unique skeleton comprising a hexahydrofuro[3,2-*b*]furan-containing heptacyclic ring system, while **55** corresponds to a loss of three carbons of the side chain of paxilline by the retro-aldol reaction. Compound **56** contains a 1,3-dioxepan-4-one ring, corresponding to a Baeyer–Villiger oxidation product of paxilline. Compound **58** derived from the cleavage of a pyrrole ring of the indole moiety. The planar structures of **54**–**58** were established by extensive analysis of 1D and 2D NMR spectra as well as high resolution mass spectrometry (HRMS). The absolute configurations of the stereogenic carbons in **54** were determined by a comparison of the calculated and experimental ECD spectra while the absolute structures of **55**–**57** were established by a comparison of their ECD curves with that of paxilline. In contrast, the absolute configurations of the stereogenic carbons in **58** were established based on a biogenic consideration [25].

Tao et al. reported the isolation of four undescribed prenylated indoles, mangrovamides D–G (**59**–**62**) (Figure 7) from the culture extract of *Penicillium* sp. SCSIO041218, which was obtained from a mangrove sediment in Sanya, China. All compounds feature a 5,8-diazatricyclo[5,2,2,0^1,5^]undecan-9-one moiety that incorporates proline (Pro). In particular, **59** and **60** contain a spiro 2-oxindole, similar to that found in **35**, **36**, **39**, and **40** (Figure 6). The structures of **59**–**62** were established by HRMS and interpretation of 1D and 2D data. The relative configurations of the stereogenic carbons in **59** were established by NOESY correlations and comparison of its ECD profile with that of mangrovamide A, which was previously isolated from the same fungus, and whose absolute structure has been established by a single-crystal X-ray analysis. The stereostructures of **60**–**62** were determined based on a comparison of their ECD profiles with that of **59** [26].

**Figure 9:**
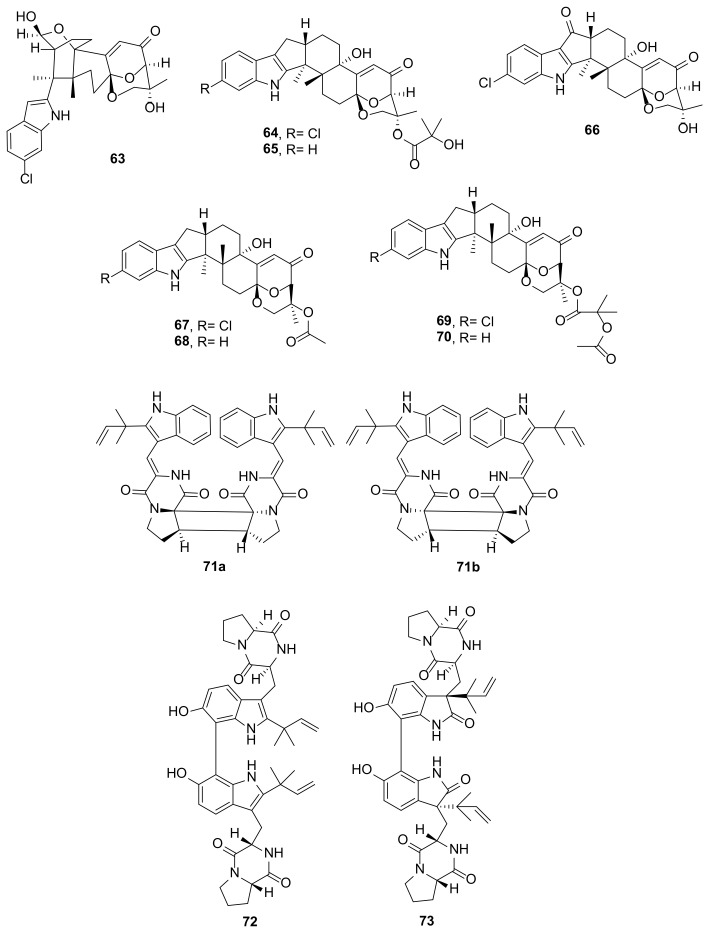
Structures of **63**–**73**.

Ascandinines A–D (**63**–**66**) (Figure 9), paxiline-like indolediterpenoids, were obtained from the culture of *Aspergillus candidus* HDN15-152, which was associated with an unidentified Antarctic sponge. Compound **63** has an unprecedented 7-chloroindole-substituted 6/6/6/6/6 pentacyclic skeleton with a 2-oxabicyclo[2.2.2]octan-3-ol motif, which is structurally derived from a cleavage of the C-17 and C-18 bond in the paxilline-like indolediterpenoids. On the other hand, **64**–**66** also share the same 2-oxabicyclo[2.2.2]octan-3-ol motif, however, they have a heptacyclic skeleton comprising the indole ring system fused with the dodecahydro-1*H*-cyclopenta[a]naphthalene ring system. The difference between **64** and **65** is the presence of a chlorine substituent in the benzene ring of the indole moiety in **64**. The relative configurations of **63**–**66** were determined by analysis of NOESY correlations and the absolute configurations of their stereogenic carbons were established by comparison of the calculated and experimental ECD spectra as well as by biogenetic considerations [27].

Asperindoles A–D (**67**–**70**) (Figure 9) are indolediterpenes with the same scaffold as those of **64**–**66**. Compounds **67**–**70** were obtained from the solid rice culture extract of *Aspergillus* sp. KMM4676, which was isolated from an unidentified colonial ascidian in Shikotan Island, Pacific Ocean. It is interesting to note that both **67** and **69** are chlorinated analogues of **68** and **70**, similar to **63** and **65**. Although chlorinated indolediterpenes are not very common, the occurrence of **63**, **67**, and **69** demonstrates that some marine-derived *Aspergillus* species have the capacity to introduce a chlorine atom to the benzene ring of the indole moiety. The structures of **67**–**70** were elucidated by extensive analysis of 1D and 2D spectral data. The relative configurations of the stereogenic carbons in **70** were established by NOESY correlations, however, their absolute configurations were proposed based on biogenetic consideration. The relative configurations of the stereogenic carbons in **69** were established by ROESY correlations and their absolute configurations were determined based on a comparison of its experimental ECD spectrum with that of **67** [28].

A pair of enantiomeric diketopiperazine prenylated indole alkaloid dimers, (−)- and (+)-asperginulin A (**71a** and **71b**) (Figure 9), was obtained as a racemic mixture from the EtOAc extract from the wheat cultures of an endophytic fungus, *Aspergillus* sp. SK-28A, which was isolated from the leaves of the mangrove plant *Kandelia candel* from the South China Sea. The structure elucidation of the compounds was carried out by a combination of extensive 1D and 2D NMR spectral analysis and X-ray diffraction analysis to obtain a planar structure and relative configurations of the compounds. By using a HPLC equipped with a chiral INA column, it was possible to separate the two enantiomers. Both enantiomers were separately recrystallized to obtain a suitable crystal for X-ray diffraction analysis using CuKα radiation to obtain crystal structures with good Flack parameters, thus allowing the determination of the absolute configurations of the stereogenic carbons of each enantiomer. The absolute configurations were also confirmed by a comparison of the calculated and experimental ECD spectra. It is interesting to note that the indole nucleus is substituted by a reverse prenyl group and a dimerization occurs by intermolecular cycloaddition of the hexahydropyrrolo [1,2-*a*]1,4-diketopiperazine moieties, forming a rare 6/5/4/5/6 pentacyclic skeleton [29].

The EtOAc extract of the rice culture of *Aspergillus austroafricanus* Y32-2, isolated from a seawater sample, which was collected from the Indian Ocean at the depth of 30 m, yielded two diketopiperazine-containing prenylated indole dimers, di-6-hydroxydeoxybrevianamide E (**72**) and dinotoamide J (**73**) (Figure 9). The structures of the compounds were elucidated by extensive analysis of 1D and 2D NMR spectral data together with HRMS. The absolute configurations of their stereogenic carbons were established by a comparison of thee calculated and experimental ECD spectra. It is interesting to note that, unlike **71b/71b**, both **72** and **73** were derived from dimerization of the activated carbon of the benzene ring of the indole moieties. However, there was a difference between the **72** and **73** resides in the indole nucleus. While **72** contains an indole nucleus substituted with a reverse prenyl group at C-2, **73** contains an oxindole whose reverse prenyl substituent is on C-3 [30].

##### Bis-/Tris-Indoles

Bis-and tris-indoles can be derived either from two (or three) direct linkage between the same indole monomers to give homodimers (or homotrimers) or different indole monomers to give heterodimers (or heterotrimers). Moreover, these indole monomers can also link together through different side chains.

**Figure 10:**
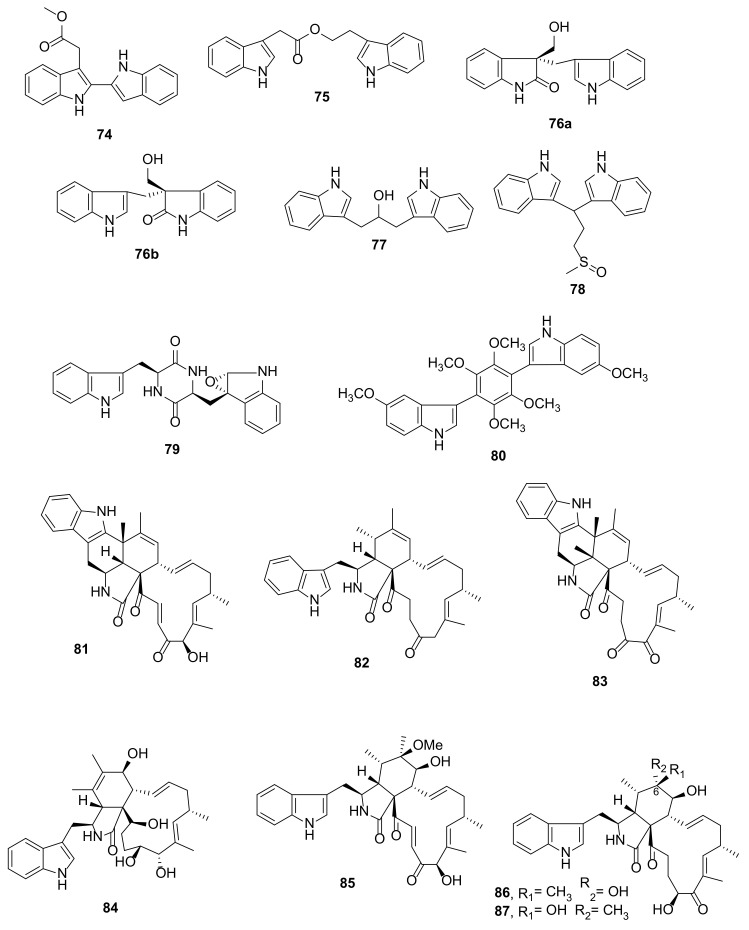
Structures of **74**–**87**.

Fusariumindole B (**74**), a bis-indole derivative comprising two indole moieties linked through C-2 and C-2′, and fusariumindole A (**75**) (Figure 10) were also isolated from the extract of the marine-derived *Fusarium* sp. L1, cultured in L-Trp-supplemented GYP medium. Compound **74** comprises two indole moieties linked through C-2 and C-2′, while in **75**, both indole moieties are linked together not directly but by an ethyl acetate ester through C-3 of the indole ring [7].

Shaker et al. [31] described the isolation of a pair of bis-indole alkaloid enantiomers (+)- and (−)-fusaspoid A (**76a** and **76b**) (Figure 10) from the extract of a marine-derived fungus *Fusarium* sp. XBB-9, obtained from the Hainan Sanya National Coral Reef Reserve, China, and cultured in a GYP medium supplemented with Phe and Trp. Compounds **76a** and **76b** were isolated as a racemic mixture whose planar structure was established by extensive 1D and 2D NMR spectral analysis as 3-(3′-methyleneindole)-hydroxymethyl-2-oxindole. The racemic mixture was separated by the HPLC chiral column into **76a** and **76b**. The stereostructure of **76a** was established by X-ray analysis as (3*S*)-(3′-methyleneindole)-hydroxymethyl-2-oxindole, whereas **76b** was identified as (3*R*)-(3′-methyleneindole)-hydroxymethyl-2-oxindole through comparison of its CD spectrum with that of **76a**.

By using GYP medium supplemented with L-Trp, L-Phe, L-Met, and L-Thr, two previously unreported bis-indole alkaloids, pseudboindoles A (**77**) and B (**78**) (Figure 10), were obtained from a culture broth of the fungus *Pseudallescheria boydii* F44-1, isolated from the inner tissue of a soft coral *Sarcophyton* sp., which was collected from the Hainan Sanya National Coral Reef Reserve, China. The planar structure of both compounds were elucidated based on HRESIMS and 1D and 2D NMR data. Compound **77** was identified as 1,3-di(1*H*-indol-3-yl)propan-2-ol, whereas **78** was identified as 3,3′[3-(methylsulfinyl)propane-1,1-diyl]bis(1*H*-indole). However, the absolute configuration of the stereogenic carbon in both compounds was not determined [32].

A diketopiperazine derivative, fellutanine A epoxide (**79**), was isolated from the culture extract of a marine sponge-associated fungus *Neosartorya glabra* KUFA 0702. The structures of **79** were established based on extensive 1D and 2D NMR spectral analysis. The absolute configurations of the stereogenic carbons of the epoxide ring (C-2′ and C-3′) were determined based on NOESY correlations, in conjunction with conformational search, molecular dynamics, and ab initio molecular modeling [13].

The EtOAc extract of the culture of *Aspergillus candidus* KUFA0062, isolated from a marine sponge *Epipolasis* sp., which was collected by scuba diving at a depth of 15–20 m from the coral reef at Similan Island National Park, Southern Thailand, produced a bis-indolyl benzenoid analogue, candidusin D (**80**) [33].

The culture extract of an endophytic fungus, *Penicillium chrysogenum* V11, isolated from the vein of a semi-mangrove tree, *Myoporum bontioides* A. Gray, which was collected in Leizhou Peninsula, China, furnished chaetoglobosin alkaloids, penochalasins I (**81**), and J (**82**) (Figure 10). Compound **81** features a 6/5/6/5/6/13 hexacyclic ring system, while **82** consists of two indole moieties, one of which is fused with a 13-membered macrocyclic ring. The structures of both compounds were elucidated by a combination of HRMS with 1D and 2D NMR spectral analysis. The absolute configurations of the stereogenic carbons were established by comparison of the calculated and experimental ECD spectra [34]. Further study of the same fungus led to the isolation of another chaetoglobosin indole alkaloid with a 6/5/6/5/6/13 hexacyclic fused ring system, named penochalasin K (**83**). The only difference between **81** and **83** is that the hydroxyl group of the 13-membered macrocyclic ring in **81** is replaced by a ketone function in **83** [35].

Cytoglobosin X (**84**) (Figure 10) was obtained from a culture extract of the endophytic fungus *Chaetomium globosum* strain E-C-2, which was isolated from the surface muscle of a sea cucumber, *Apostichopus japonicus*, collected from Chengshantou Island, Weihai City, the Yellow Sea, China [36]. The culture of a coral-associated fungus *C. globosum* C2F17, isolated from a coral *Pocillopora damicornis*, which was collected from the seashore near Sanya Bay, Hainan Province, China, yielded a cytochalasan alkaloid 6-*O*-methylchaetoglobosin Q (**85**) (Figure 10). The structure of **85** was determined using extensive spectroscopic analysis whereas the configurations of its stereogenic carbons were determined through a comparison of the calculated and experimental ECD spectra. The authors speculated that **85** was probably an artifact derived from acidic solvolysis of the previously reported epoxide of chaetoglobosin A [37]. Cytoglobosin H (**86**) and its C-6 epimer, cytoglobosins I (**87**) (Figure 10) were obtained from the extract of a solid rice culture of *C. globosum*, which was isolated from deep-sea sediments collected from the Indian Ocean. The structures of both compounds were established by extensive 1D and 2D NMR spectral analysis. Their relative configurations were determined by ROESY correlations, however, the absolute configurations of their stereogenic carbons were not determined [38].

##### Annelated Indoles

**Figure 11:**
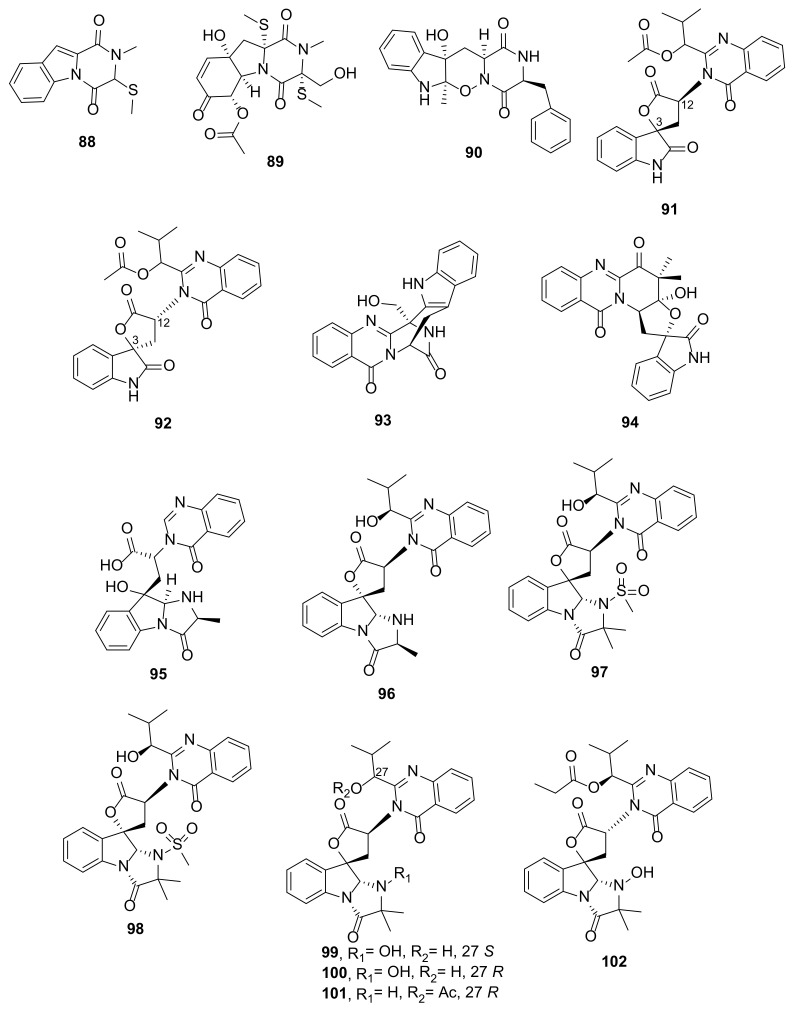
Structures of **88**–**102**.

Two methylsulfinyldiketopiperazine-containing annelated indoles, dichocerazines A (**88**) and B (**89**) (Figure 11), were isolated from the culture extract from a soft coral-associated fungus *Dichotomomyces cejpii* F31-1, cultured in a GYP medium supplemented with L-Trp and L-Phe. The structures of **88** and **89** were elucidated by the analysis of 1D and 2D NMR spectra as well as HRMS data. Compound **88** was isolated as a racemate since it showed no optical rotation, and an effort to separate the enantiomers by a HPLC equipped with a Chiralcel OD column was not successful. In contrast, **89** was isolated as a pure compound and the absolute configurations of its stereogenic carbons were established by comparison of the calculated and experimental ECD spectra [8]. Structurally, **88** was derived from a condensation of Trp and Gly whereas **89** was derived from a condensation of Trp and Ser, and methylsulfinyl substituents were introduced in a later stage to the carbon adjacent to the carbonyl function.

A diketopiperazine-containing oxindole alkaloid, raistrickindole A (**90**) (Figure 11), was obtained from the EtOAc extract from a solid rice culture of *Penicillium raistrickii* IMB17-034, which was isolated from marine sediments collected in a mangrove swamp in Sanya, Hainan Province, China. Structurally, **90** was derived from a condensation of Trp and Phe, therefore the absolute configuration of L-Phe was established by the advanced Marfey’s analysis. The absolute configurations of the rest of the stereogenic carbons were determined by comparison of the calculated and experimental ECD spectra [39].

The EtOAc extract of a culture broth of *Aspergillus* sp. HNMF114, isolated from a bivalve mollusk *Sanguinolaria* *chinensis*, which was collected from Haikou Bay, Hainan Province, China, furnished aspertoryadins F (**91**) and G (**92**) (Figure 11). Compounds **91** and **92** contain 2-indolone moiety linked to a quinazolinone ring system through a five-membered spiro lactone. The planar structures of the compounds were elucidated by analysis of 1D and 2D spectra as well as HRMS data. However, the ECD spectra of **91** and **92** are mirror images through the Cotton effects (CEs) around 210 and 230 nm. According to the ECD exciton coupling rule, the absolute configurations of C-3 and C-12 of **91** and **92** were determined as 3*S*, 12*S*, and 3*R*, 12*R*, respectively. However, the absolute configuration of the stereogenic carbon in the side chain of the quinazolinone moiety was the same [40].

Fumigatoside E (**93**) (Figure 11), whose indole moiety is fused with a pyrazinone ring of the pyrazinoquinazolinone moiety, was obtained from a culture extract of *Aspergillus fumigatus* SCSIO 41012 isolated from deep-sea sediments that were collected from the Indian Ocean. The planar structure of the compound was elucidated by HRMS and extensive 1D and 2D NMR spectral analysis. The absolute configurations of its stereogenic carbons were established as 3*R*, 14*R* by comparison of the calculated and experimental ECD spectra [41].

Scedapin E (**94**) (Figure 11), an indole alkaloid comprising an oxindole moiety linked to a pyrazinoquinazolinone moiety via a spiro pyran ring, was isolated from the extract of a marine-derived fungus *Scedosporium apiospermum* F41−1, cultured in a GYP medium supplemented with L-Trp, L-Phe, L-Thr, and D,L-Met. The planar structure of **94** was elucidated by HRMS and 1D and 2D NMR spectral analysis. The stereostructure of **94** was established by a single-crystal X-ray analysis [12].

Fumigatoside F (**95**) (Figure 11), an indole alkaloid comprising a *6-5-5*-imidazoindolone ring linked to a quinazolinone ring system via an ethylene bridge, was obtained from a culture extract of *A. fumigatus* SCSIO 41012, isolated from deep-sea sediments that were collected from the Indian Ocean. The planar structure of the compound was established by 1D and 2D NMR spectral analysis as well as HRMS data. The proton chemical shift values suggest that the structure of **95** was derived from the opening of the lactone ring in tryptoquivaline E, however, the absolute configurations of its stereogenic carbons were not determined [41].

Aspertoryadins I (**96**) and J (**97**) (Figure 11) were isolated from the culture extract of the fungus *Aspergillus* sp. HNMF114, obtained from a mollusk *Sanguinolaria chinensis*. These alkaloids comprise an indole moiety fused with an imidazolone ring to form a *6-5-5*-imidazoindolone ring system, which is linked to a quinazolinone ring system through a five-membered spiro lactone such as in tryptoquivalines [42]. Aspertoryadins A−E (**98**–**102**) (Figure 11), which also contain an imidazoindolone moiety linked to a quinazolinone ring system through a five-membered spiro lactone, were also isolated from the same fungus. Compound **98** has an unusual methylsulfonyl group attached to the nitrogen atom of the imidazolone moiety while the rest of the compounds, except for **101**, has a hydroxyl group on this nitrogen atom. The stereostructure of **98** was established by X-ray analysis and was confirmed by the comparison of the calculated and experimental ECD spectra. Compounds **99** and **100** are C-27 epimers whose absolute configurations of C-27 were determined by comparison of their C-27 chemical shift values with that of **98**. The relative configurations of **101** and **102** were established based on ROESY correlations, however, the absolute configurations of their stereogenic carbons were not determined [40].

**Figure 12:**
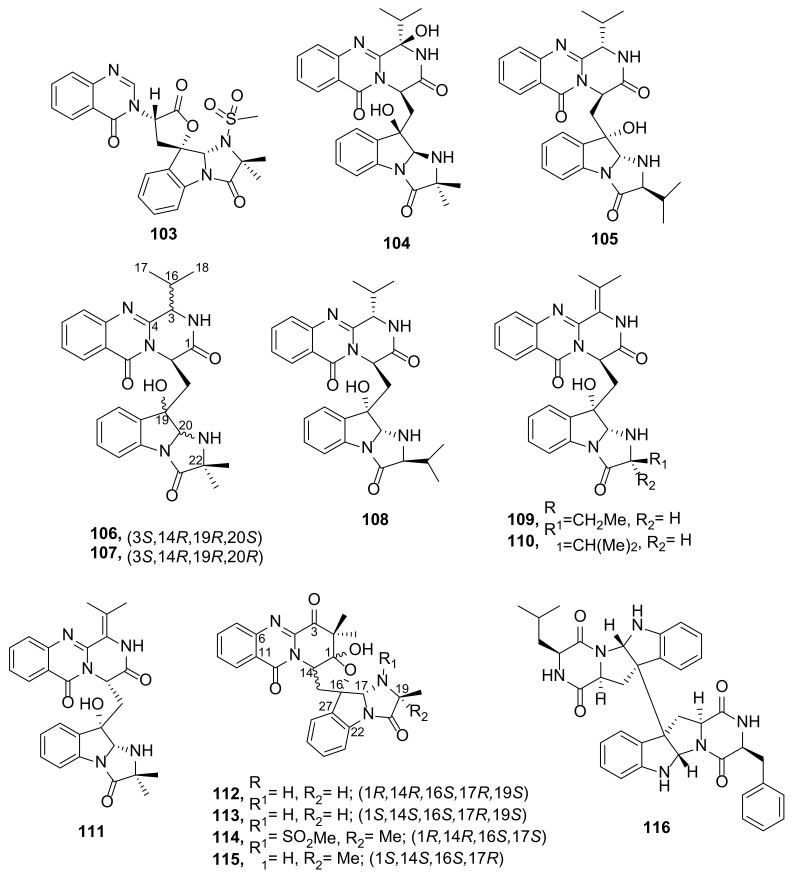
Structures of **103**–**116**.

Scetryptoquivaline A (**103**), an *N*-methylsulfonyl imidazoindolone connected to quinazolinone moiety through a five-membered lactone, and scequinadoline I (**104**) (Figure 12), an imidazoindolone connected to an isopropyl pyrazinoquinazolinone moiety through a methylene bridge (Figure 12), were also isolated from the culture extract of a coral-associated fungus *Scedosporium apiospermum* F41-1 [11] while aspertoryadin H (**105**), another imidazoindolone connected to isopropylquinazolinone moiety through a methylene bridge, was also isolated from a mollusk-associated fungus *Aspergillus* sp. HNMF114 [42]. Scequinadolines A−F (**106**–**111**) (Figure 12), comprising an imidazoindolone connected to an isopropylpyrazinoquinazolinone moiety through a methylene bridge, and scedapins A-D (**112**–**115**) (Figure 12), whose imidazoindolone moiety is connected to a pyrazinoquinazolinone through a spiro tetrahydrofuran, were also isolated from the culture extract of a soft coral-associated fungus *S. apiospermum* F41-1 [12].

A heterodimer of an indolodiketopiperazine derivative, named SF5280-415 (**116**) (Figure 12) was obtained from the culture extract of *Aspergillus* sp. SF-5280, isolated from an unidentified sponge that was collected at Cheju Island, Korea. The structure of the compound was established by a combination of HRMS and 1D and 2D NMR spectral analysis. The configurations of the amino acid constituents, Phe and Leu, were determined by Mafrey’s method while the relative configurations of the stereogenic centers of the diketopiperazine ring and C-2 and C-3 of the indole moiety were established by observation of NOESY correlations and compared with those of the previously described compounds (WIN 64745 and ditryptophenaline) [43].

#### 2.1.2. Marine-Derived Bacteria

In comparison with marine-derived fungi, marine-derived bacteria are less prolific in terms of indole alkaloid production. Most of the indole alkaloids discovered in this period are simple, bis-, and tris-indoles.

##### Simple Indoles

**Figure 13:**
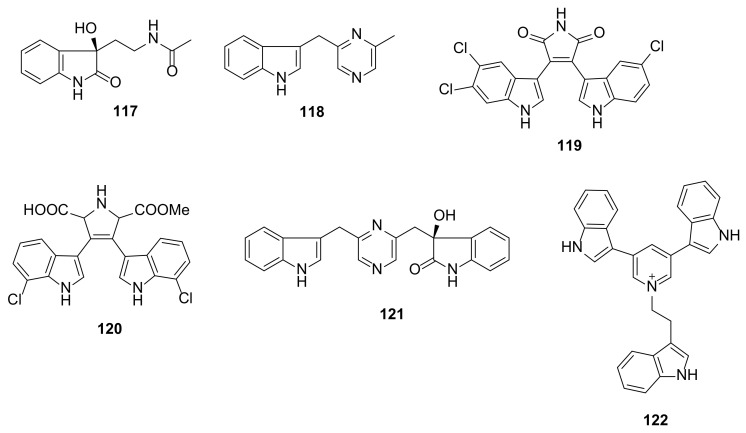
Structures of 117–122.

A simple indole alkaloid comprising an oxindole with an amide side chain, named bacilsubteramide A (**117**) (Figure 13), was isolated from an EtOAc extract of the culture of *Bacillus subterraneus* 11593, which was collected from the South China Sea with the depth of 2918 m. The structure of **117** was elucidated by 1D and 2D NMR spectral analysis while the absolute configuration of its stereogenic carbon was established by a comparison of the calculated and experimental ECD spectra [44]. Indolepyrazine B (**118**) (Figure 13) is a pyrazine-containing simple indole, obtained from an extract of a marine-derived *Acinetobacter* sp. ZZ1275, which was isolated from a mud sample collected from the coastal area of Karachi, Sindh, Pakistan [45].

##### Bis-/Tris-Indoles

A culture extract of *Streptomyces* sp. SCSIO 11791, isolated from a sediment sample collected from the South China Sea at a depth of 1765 m yielded two chlorinated bis-indole alkaloids, dienomycin (**119**) and 6-methoxy-7′,7″-dichlorochromopyrrolic acid (**120**) (Figure 13). Compound **119** contains two indole moieties linked together through 1*H*-pyrrole-2,5-dione, while the two indole moieties in **120** are linked through dimethyl 2,5-dihydro-1*H*-pyrrole-2,5-dicarboxylate. The structures of both compounds were elucidated by analysis of HRMS and 1D and 2D NMR spectra [46].

Besides the simple indole **118**, the culture of a marine-derived *Acinetobacter* sp. ZZ1275 also furnished a bis-indole alkaloid indolepyrazine A (**121**) (Figure 13) comprising one indole and one oxindole ring system linked by a 2, 2-dimethylpyrazine unit. The structure of **121** was elucidated by HRMS and 1D and 2D NMR spectral analysis. The configuration of the stereogenic carbon on the oxindole moiety was established through a comparison of the calculated and experimental ECD spectra [45].

Tricepyridinium (**122**) (Figure 13), a novel pyridinium with three indole moieties, was isolated from the culture extracts of the *Escherichia coli* transfected by metagenomic DNA prepared from the marine sponge *Discoderma calyx* collected from Shikine-jima Island in Japan. The structure of **122** was elucidated by a combination of HRMS with 1D and 2D NMR spectral analysis [47].

### 2.2. Marine Invertebrates

#### 2.2.1. Marine Sponges

Marine sponges are also a source of structurally diverse indoles. However, only simple indoles and bis-/tris-indoles were reported from marine sponges in this period.

##### Simple Indole Alkaloids

**Figure 14:**
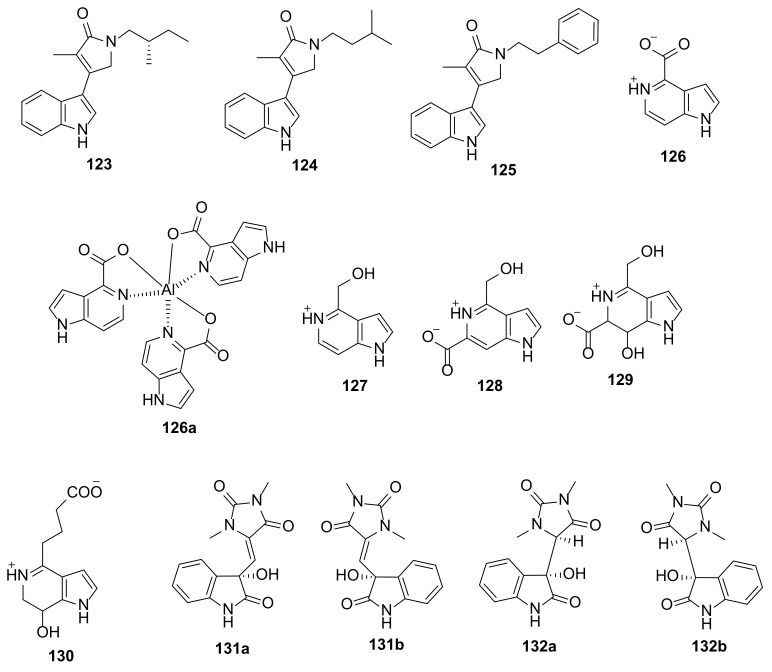
Structures of **123**–**132**.

Simple indole alkaloids consisting of an indole nucleus linked to a *N*-substituted-γ-lactams, named psammocindoles A–C (**123**–**125**) (Figure 14), were isolated from a marine sponge *Psammocinia vermis* (order Dictyoceratida, family Irciniidae) collected from Chuja-do, Korea. The structures of **123**–**125** were elucidated based on a combination of a high-resolution fast atom bombardment mass spectrometry (HRFABMS) and 1D and 2D spectral analysis. In the case of **123**, the absolute configuration of the stereogenic carbon was determined by a comparison of its optical rotation with that of a synthetic version [48].

The 5-azaindole derivatives, guitarrins A–E (**126**–**130**) and an aluminum complex compound, aluminumguitarrin A (**126a**), (Figure 14), were isolated from the marine sponge *Guitarra fimbriata.* Compounds **126**–**129** and **126a** were isolated from the sample of *G. fimbriata*, collected by dredging near Chirpoy Island in the Pacific Ocean, while **130** was isolated from a sample of the same sponge but collected near Urup Island, Sea of Okhotsk. The structures of all the isolated compounds were elucidated by HRMS and 1D and 2D NMR spectral analysis. In the case of **126** and **126a**, their structures were confirmed by a single-crystal X-ray diffraction analysis [49].

**Figure 15:**
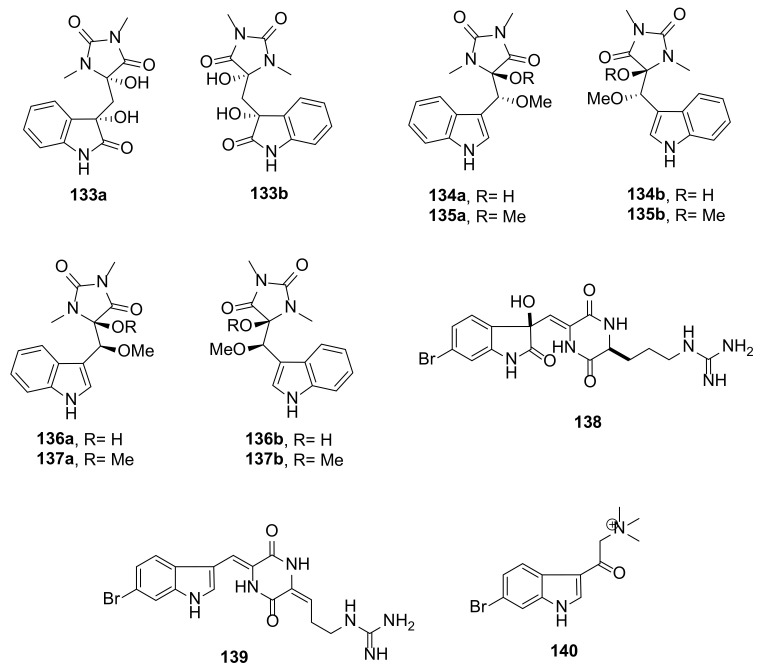
Structures of **133**–**140**.

Seven pairs of oxygenated aplysinopsin-type enantiomers, (+)- and (−)-oxoaplysinopsins A–G (**131**–**137**) (Figure 14 and Figure 15) were isolated from a marine sponge *Fascaplysinopsis reticulata*, which was collected from Xisha Island in the South China Sea. The enantiomers were separated by a chiral HPLC equipped with a chiral analytical column (CHIRALPAK IC column). The planar structures of all compounds were determined by extensive analysis of NMR spectroscopic and HRMS data while the absolute configurations of the stereogenic carbons in the compounds were established by a comparison of their calculated and experimental ECD spectra [50].

An alkyl-guarnidine-substituted diketopiperazine-containing bromooxindole, geobarrettin A (**138**), an alkylguarnidine-substituted diketopiperazine-containing bromoindole, geobarrettin B (**139**), and geobarrettin C (**140**) (Figure 15), a trimethyloxoammonium-containing bromoindole, were isolated from a sub-Arctic sponge *Geodia barretti* collected from the west of Iceland. The structures of **138**–**140** were elucidated by interpretation of HRMS and 1D and 2D NMR spectral data. The configuration of C-3 of the oxindole moiety in **138** was determined by a comparison of the ECD spectra of the hydrolysis product of **138** and of (*R*)-3-proplyldioxindole, while the absolute configuration of C-12 of the diketopiperazine ring was determined by Marfey’s method [51].

##### Bis-/Tris-Indole Alkaloids

**Figure 16:**
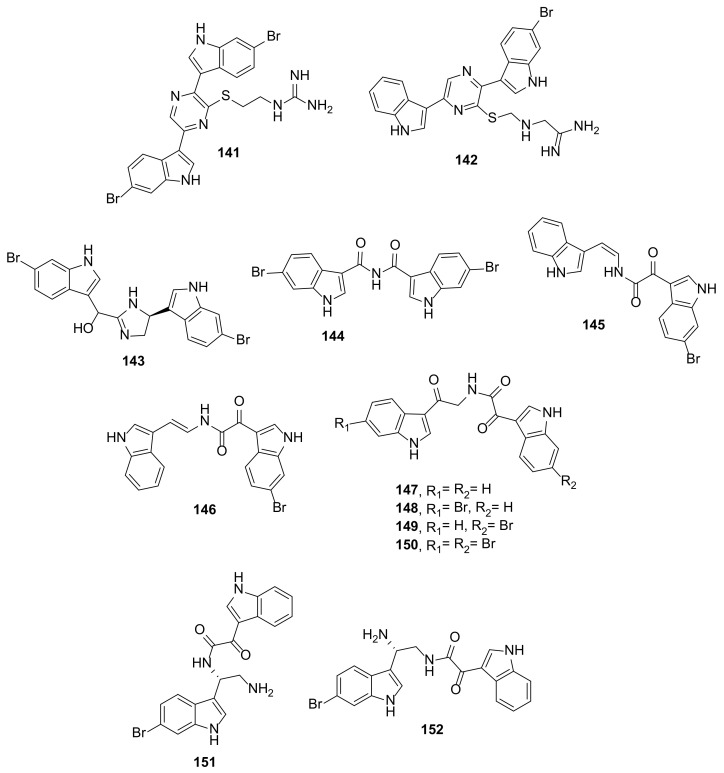
Structures of **141**–**152**.

Dragmacidin G (**141**) (Figure 16), a bis-bromoindole linked by a pyrazine ring with a *N*-(2-mercaptoethyl)-guanidine side chain, was isolated from the marine sponge of an unidentified species of *Spongosorites* (Family Halichondriidae) collected from Long Island, Bahamas by the Johnson-Sea-Link I manned submersible at a depth of 630 m. The structure of the compound was elucidated by extensive analysis of NMR spectra and HRMS [52]. Compound **141** was also isolated, together with dragmacidin H (**142**) (Figure 16), from a marine sponge *Lipastrotethya* sp., which was collected by dredging at Kurose, north of Hachijo Island, Japan [53]. Compound **142** differs from **141** in that only one indole moiety is brominated and the side chain of the pyrazine ring is *N*-(2-mercaptomethyl)-guanidine instead of *N*-(2-mercaptoethyl)-guanidine.

Dihydrospongotine C (**143**) (Figure 16), a member of bis-indole alkaloids comprising two bromoindole moieties linked together by 4,5-dihydro-1*H*-imidazol-2ylmethanol, was isolated from a deep-sea sponge *Topsentia* sp. collected in Palau at the depth of 140 m. The structure of the compound was established by extensive analysis of 1D and 2D spectra and HRMS data. The absolute configurations of the stereogenic carbons were established by a comparison of the calculated and experimental ECD spectra as 4*S*, 6*S* [54].

Splendamide (**144**) (Figure 16), a bis-bromoindole-3 carboxamide, was isolated from a marine sponge *Jaspis splendens*, which was collected by scuba diving (−23 m) at Mid Reef, Great Barrier Reef, North Queensland, Australia. Indole-3-carboximidamides are rarely encountered in nature and only a few examples have been reported [55].

*Cis/trans* isomers of bis-indoles connected through an α-ketovinylamide moiety, named (*Z*)-coscinamide D (**145**) and (*E*)-coscinamide D (**146**) (Figure 16), were isolated together with four bis-indoles linked through a diketoamide moiety, lamellomorphamides A–D (**147**–**150**) (Figure 16), from a marine sponge *Lamellomorpha strongylata,* which was collected from the Western Continental Slope (Station J954), Northland, New Zealand, at a depth of 200 m. Although **147**–**150** were new natural products, they had been previously reported as intermediates in the synthesis of 6′, 6”-didebromo-*cis-*3,4-dihydrohamacanthin B, 6′-debromo-*cis*-3,4-dihydrohamacanthin B, and hamacanthin analogues [56].

Calcicamides A (**151**) and B (**152**) (Figure 16), two bis-indole alkaloids comprising one indole and one 6-bromoindole connected through an aminoalkyl α-ketoamide moiety, were isolated from a marine sponge *Spongosorites calcicole* collected at Rathlin Island (Co. Antrim), Northern Ireland, at a 18 m depth. Compounds **151** and **152** are constitutional isomers as they have the same molecular formula. 1D and 2D-NMR spectral analysis revealed that both compounds have an indole portion linked to the α-ketoamide moiety that is connected to C-8 and C-9 of 6-bromotryptamine in **151** and **152**, respectively. The absolute configuration of the stereogenic carbon (C-8) of both compounds was established as *S* through a comparison of the calculated and experimental ECD spectra. The structures of **151** and **152** were hypothesized as intermediates in the biosynthesis of a cyclic structure that linked to both indole moieties such as spongotine and toptensin as well as the hamacanthin B derivative [57].

**Figure 17:**
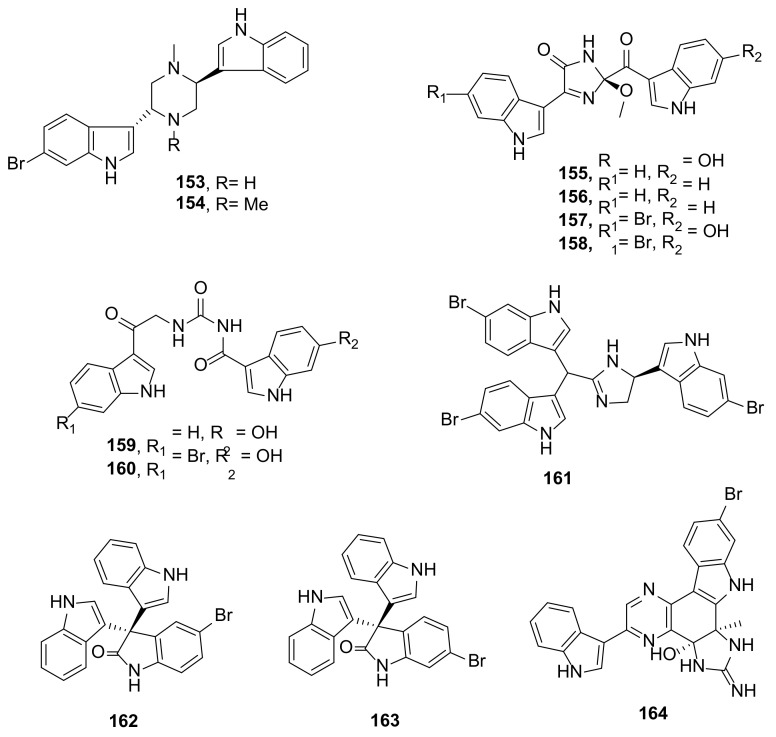
Structures of **153**–**164**.

Dragmacidins I (**153**) and J (**154**) (Figure 17), bis-indole alkaloids featuring one indole and one 6-bromoindole linked to C-2 and C-5 of a piperazine ring, were isolated from a marine sponge *Dragmacidon* sp., which was collected from Kashani Island in Tanzania. The absolute configurations of the stereogenic carbons in **153** and **154** were proposed to be 2*R*,5*S* on the basis of their measured optical rotation values, which were very close to those reported for dragmacidin A, prepared by enantioselective total synthesis [58].

Spongosoritins A–D (**155**–**158**) (Figure 17), bis-indole alkaloids comprising two indole moieties linked through a 2-keto-2-methoxy-1-imidazole-5-one core, along with spongocarbamides A (**159**) and B (**160**) (Figure 17), another two bis-indoles whose two indole moieties were connected through a linear type keto urea, were isolated from a marine sponge *Spongosorites* sp. collected at the depth of 25–30 m offshore Seogwipo, Jeju Island, Korea. The structures of **155**–**160** were elucidated by a combination of HRMS and 1D and 2D NMR spectral analysis. The absolute configuration of the stereogenic carbon in the imidazolone ring of **155**–**158** was established by comparison of the calculated and experimental ECD spectra [59].

Tulongicin A (**161**) (Figure 17) is a tris-indole alkaloid featuring an imidazole ring connected to three 6-bromoindole units. One of the 6-bromoindole is directly linked to C-4 of the indole core while another two 6-bromoindole units formed a bis(indolyl) methane moiety and linked to C-2 of the imidazole ring. Compound **161** was isolated together with **141** from a deep-water marine sponge *Topsentia* sp. [54].

5-Bromotrisindoline (**162**) and 6-bromotrisindoline (**163**) (Figure 17) are tris-indole alkaloids comprising two indoles and one bromooxindole linked together through C-3 of each indole unit. The difference between **162** and **163** is that the bromine atom is on C-5 of the oxindole ring in **162**, but on C-6 in **163**. Although **162** and **163** were first isolated as new natural products from a marine sponge *Callyspongia siphonella* collected from Hurghada, Egypt, along the Red Sea Coast, they have already been reported as synthetic intermediates [60].

A bis-indole alkaloid, myrindole A (**164**) (Figure 17), was isolated from a marine sponge *Myrmekioderma* sp. collected in Sagosone, Japan. Compound **164** features a tetrahydroquinoxaline ring system fused with imidazolidin-2-imine as a central core, having the cyclohexane ring fused with 6-bromoindole while C-2 of the pyrazine ring is linked to an indole moiety. Besides the usual 1D and 2D NMR spectral analysis, ^1^H-^15^N HMBC and 1,n-ADEQUATE correlations were used to unravel this complex structure. The absolute configurations of C-5 and C-6 in **164** were determined as 5*S*,6*R* by comparison of the calculated and experimental ECD spectra [61].

#### 2.2.2. Bryozoans

Bryozoans are another rich source of indole alkaloids. Simple, prenylayed, and annelated indoles have been reported from bryozoans.

##### Simple Indole Alkaloids

**Figure 18:**
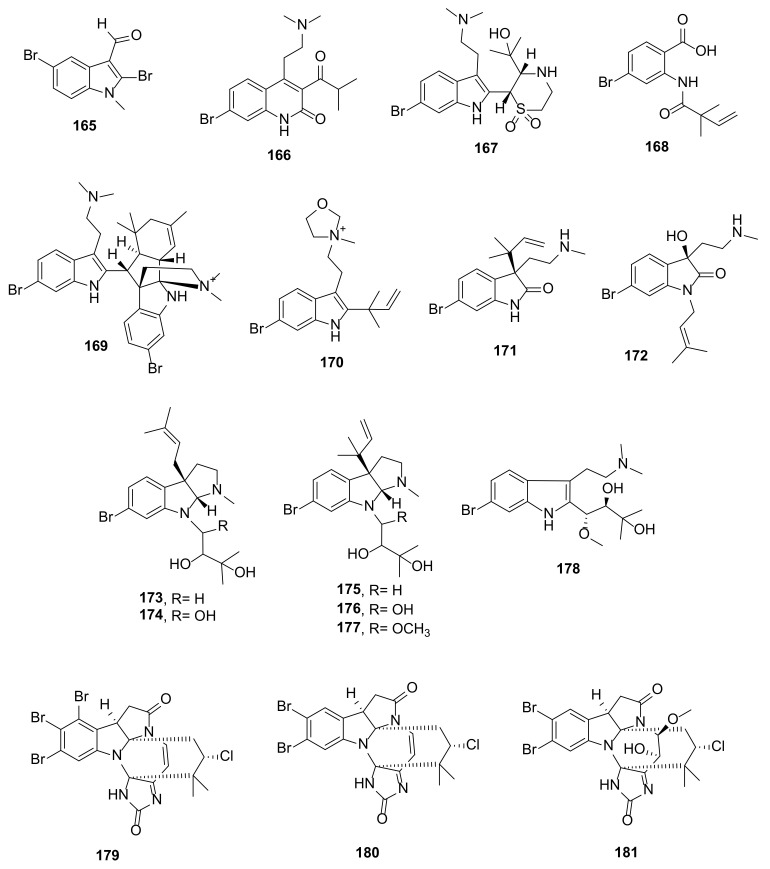
Structures of **165**–**181**.

During a high-throughput screening of an Australian marine invertebrate extract library against *Plasmodium falciparum*, Kleks et al. [62] isolated 2,5-dibromo-1-methyl-1*H*-indole-3-carbaldehyde (**165**) (Figure 18) from samples of a bryozoan *Amathia lamourouxi*, collected from the rock pools of Woolgoolga and as storm debris from Korora Beach, Coffs Harbour, New South Wales, Australia.

##### Prenylated Indoles

The organic extract of a marine bryozoan *Flustra foliacea*, collected near the southwest coast of Iceland at 13 m depth, furnished 13 prenylated indole alkaloids, *viz.* flustramines Q–W (**166**–**172**) and flustraminols C−H (**173**–**178**) (Figure 18). Although the structures of **166** and **168** apparently do not contain the indole ring system, they were derived from the degradation and rearrangement of the indole nucleus. Compound **167** shows interesting features with *N,N*-dimethylaminoethyl and thiomorpholine 1,1-dioxide ring substituents, while **169** is a bis-indole with one of the indole rings fused with a prenyl-derived bicyclic system. Compounds **175**–**177** are diprenylated pyrrolidine-fused indoles. The structures of the compounds were elucidated by extensive analysis of 1D and 2D NMR spectra and HRMS data. In the case of **167**, the absolute configurations of the stereogenic carbons were determined by a comparison of the calculated and experimental ECD spectra of its *bis-*trifluoroacetate double salt. The absolute configurations of the stereogenic carbons in **171, 172**, **174**, **175**–**178** were established based on a comparison of the CEs of their ECD spectra with those of the compounds containing related chromophores and whose absolute structures have been established. Only relative configurations of the stereogenic carbons of **173** and **178** were determined by NOESY correlations and by observation of the ^1^H chemical shift values, respectively [63].

##### Annelated Indole Alkaloids

Securamines H–J (**179**–**181**) (Figure 18), hexacyclic annelated indole alkaloids featuring an imidazolone-containing bromoindole fused with pyrrolidinone moiety, were isolated from the Arctic bryozoan *Securiflustra securifrons*, collected by an Agassiz trawl at 73 m depth from west Spitzbergen. The structures of the compounds were established by extensive analysis of 1D and 2D NMR spectra and HRMS data, however, the absolute configurations of their stereogenic carbons were not determined [64].

### 2.3. Marine Plants

Marine plants seem to not be very good producers of indole alkaloids. Only one marine alga and one mangrove plant have been reported as sources of indole alkaloids during this period.

#### 2.3.1. Algae

**Figure 19:**
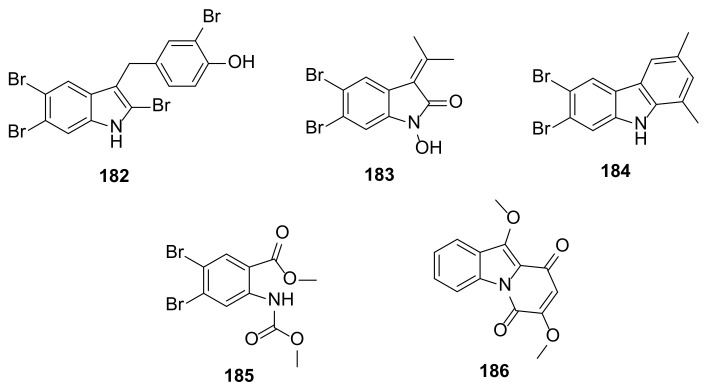
Structures of **182**–**186**.

Four brominated indoles (**182**–**185**) (Figure 19) were isolated from the organic extract of the marine alga *Laurencia similis* collected in the South China Sea. Compound **182** is a simple dibromoindole with a benzyl group on C-3 of the indole core while **183** is an oxindole with a 2-propylidene on C-3. On the other hand, **184** is an annelated bromoindole while **185** features a carbamate-containing benzoic acid derivative resulting from the oxidative ring opening of a pyrrole ring of the indole nucleus [65].

#### 2.3.2. Mangrove Trees

An annelated indole, acanthiline A (**186**) (Figure 19), featuring a pyrido[1,2-*a*]indole skeleton, was isolated from a mangrove tree *Acanthus ilicifolius* Linn, collected at the Zhanjiang Mangrove National Nature Reserve, Guangdong Province, China [66].

## 3. Biological and Pharmacological Activities of Indole Alkaloids

### 3.1. Antimicrobial Activity

Although the prenylated indoles, 2-(2-methyl-3-en-2-yl)-1*H*-indole-3-carbaldehyde (**9**) and 2-(2,2-dimethylcyclopropyl)-1*H*-indole-3-carbaldehyde (**10**) (Figure 4) did not show antibacterial activity against either Gram-negative (*Escherichia coli* ATTC 25922 and *Pseudomonas aeruginosa* ATTC 27853) or methicillin-resistant *Streptococcus aureus* (MRSA) and vancomycin-resistant enterococci (VRE), both compounds showed inhibition of biofilm production in *S. aureus* ATCC 25923, while only **10** significantly reduced biofilm production by *E. coli* ATCC 25922. Moreover, **9** and **10** induced a weak increase in the halo of a partial inhibition of vancomycin in VRE *Enterococcus faecalis* B3/101 when compared to vancomycin alone, whereas **9** showed a modest synergistic effect with cefotaxime in the impregnated disks against extended spectrum β-lactamase (ESBL) *E. coli* strain SA/2 [14].

The prenylated indoles, asperthins A–F (**41**–**46**) (Figure 6), were evaluated for their activities against agricultural pathogenic bacteria and fungi. Asperthin A (**41**) exhibited both antibacterial and antifungal activities with MIC values of 50, 12.5, and 100 µg/mL against the bacteria *Xanthomonas oryzae pv. oryzae* and *X. oryzae pv. oryzicola,* and the fungus *Rhizoctonia solani*, respectively. Moreover, **41** also showed moderate antibacterial activity against four fish pathogens, *viz. Edwardsiella tarda*, *Vibrio anguillarum, V. parahaemolyticus*, and *Aeromonas hydrophilia*, with MIC values of 16, 8, 16, and 32 µg/mL, respectively. Except for *X. oryzae pv. oryzicola,* **41** showed higher MIC values against all the tested bacteria (MIC of chloromycetin, a positive control, against *X. oryzae pv. Oryzae*, *X. oryzae pv. Oryzicola*, *E. tarda*, *V. anguillarum, V. parahaemolyticus*, and *A. hydrophilia* = 12.5, 12.5, 2, 0.5, 2 and 2 µg/mL, respectively). On the other hand, asperthin E (**45**) only showed weak antifungal activity against *R. solani* with a MIC value of 25 µg/mL (MIC of a positive control, ketoconazole = 0.78 25 µg/mL) [22].

Penochalasins I (**81**), J (**82**) and K (**83**) (Figure 10) were evaluated for the in vitro antifungal activity against plant pathogenic fungi *viz*. *Colletotrichum gloeosporioides*, *R. solani*, *Colletotrichum musae*, and *Penicillium italicum*. Compound **81** was inactive against all the pathogenic fungi while **82** showed moderate activity against *C. gloeosporioides* (MIC = 25.08 µM) and *R. solani* (MIC = 50.17 µM) and **83** exhibited more potent activity against *C. gloeosporioides* (MIC = 6.13 µM) and *R. solani* (MIC = 12.26 µM), respectively. Both **82** and **83** showed MIC values lower than a positive control, carbendazim, against *C. gloeosporioides* and *R. solani* (MIC values of 65.38 and 32.69 µM, respectively [34,35].

The quinazoline-containing indoles, aspertoryadins F (**91**) and G (**92**) (Figure 11) were evaluated for their antibacterial activity against *S. aureus*, *E. coli*, *Bacillus subtilis*, and *Streptococcus agalactiae* as well as quorum sensing (QS) inhibitory activity against *Chromobacterium violaceum* CV026. Both compounds were found to be void of antibacterial activity but exhibited QS inhibitory activity against *C. violaceum* CV026 with MIC values of 32 µg/well whereas the positive control, bromofuranone C30, exhibited a MIC value of 1 µg/well [40].

Fumigatoside E (**93**) (Figure 11) showed antibacterial activity against Gram-negative bacteria *Acinetoobacter baumannii* ATCC 19606, *A. baumannii* ATCC 15122, and *Klebsiella pneumoniae* ATCC 14578, and a Gram-positive bacterium, *S. aureus* ATCC 16339 as well as two strains of fungi *Fusarium oxysporum* f. sp. *cucumerinum* and *F. oxysporum* f. sp. *momordicae.* Compound **93** exhibited moderate to strong activity, with MIC values of 6.25 µg/mL against *A. baumannii* ATCC 15122 and *S. aureus* ATCC 16339, and 12.5 µg/mL against *A. baumannii* ATCC 19606 and *K. pneumoniae* ATCC 14578. Interestingly, the MIC value of **93** against *A. baumannii* ATCC 15122 was half the MIC value of a positive control, streptomycin (MIC = 12.5 µM) while fumigatoside F (**88**) only exhibited moderate antibacterial activity against *A. baumannii* ATCC 19606, with a MIC value of 6.25 µM (streptomycin showed MIC value = 1.565 µM). In contrast, **93** exhibited weak activity against *F. oxysporum* f. sp. *cucumerinum* (MIC = 25 µM) but strong activity against *F. oxysporum* f. sp. *momordicae* (MIC = 1.565 µM) when compared to nystatin, a positive control, whose MIC value was 12.5 µM [41].

The bis-indole alkaloids, dienomycin (**119**) and 6-methoxy-7′,7″-dichorochromopyrrolic acid (**120**) (Figure 13), were evaluated for antibacterial activity by a disk diffusion method against Gram-positive bacteria including *Micrococcus luteus* ML01, *S. aureus* ATCC 29213, and a panel of MRSA isolated from human patients (MRSA 991, MRSA 1862, MRSA 669 A, and MRSA A2) and pig (MRSA GDQ6P012P and MRSA GDE4P037P) as well as Gram-negative bacteria including *A. baumannii* ATCC 19606, *Vibrio coralliilyticus* ATCC BAA-450, and *V. alginolyticus* XSBZ14, however, **119** and **120** showed growth inhibition of Gram-positive bacteria, but not Gram-negative bacteria, at 10 μg per filter paper. Determination of MIC by a broth microdilution method revealed that **119** was many folds more active than **120** toward *M. luteus* ML01, *S. aureus* ATCC 29213, and all the MRSA strains, especially *M. luteus* ML01 (MIC = 0.5 µg/mL) and *S. aureus* ATCC 29213 (MIC = 1 µg/mL). Moreover, **119** displayed lower MIC value than kanamycin but higher MIC value than vancomycin, which were used as positive controls against these microorganisms. Structure–activity relationship (SAR) study revealed that the presence of a chlorine atom on C-6” of the indole moiety played a crucial role in the antibacterial activity of this series of compounds [46].

Pyrazine-containing indolepyrazines A (**121**) and B (**118**) (Figure 13) showed potent antimicrobial activities against methicillin-resistant *S. aureus* (MRSA), *E. coli*, and *Candida albicans* with MIC values of 12 µg/mL, 8–10 µg/mL, and 12–14 µg/mL, respectively. However, the MIC values of both **118** and **121** were several folds higher than the positive controls, gentamicin for antibacterial, and amphotericin B for antifungal activities. Interestingly, the absence of 1-hydroxy-1,3-dihydro-2*H*-indol-2-one moiety in **118** did not affect the potency of antibacterial and antifungal activities on the tested strains [45].

A bromide salt of a synthetic version of tricepyridinium (**122**) (Figure 13) was found to display a significant antibacterial activity against *Bacillus cereus* with a MIC value of 0.78 μg/mL and methicillin-sensitive *S. aureus* (MSSA) with a MIC value of 1.56 μg/mL as well as a moderate antifungal activity against *C. albicans* with a MIC value of 12.5 μg/mL. However, the compound did not show any activity against *E. coli* at 100 μg/mL. Structure–activity relationship study with a synthetic version and two indole moieties revealed that the three indole moieties of **122** and the charged pyridinium were mainly responsible for a strong cytotoxicity toward Gram-positive bacteria and fungi. Moreover, it was found that the indole group at the 3- or 5-position was more important than an indole group at the 8″-position [47].

A pyrazino bis-indole dragmacidin G (**141**) (Figure 16) was found to exhibit a potent antibacterial activity against *S. aureus* and MRSA with MIC values of 0.62 µg/mL (1 µM) for both strains. Compound **141** also displayed an anti-mycobacterial activity in the assay that used *Mycobacterium tuberculosis* CDC1551 carrying the pMV306hsp-LuxG13 integrative plasmid, which provides constitutive expression of the *luxCDABE* operon [52].

The bis-indoles, spongosoritins A–D (**155**–**158**) and spongocarbamides A (**159**) and B (**160**) (Figure 17) were assayed for their antibacterial activity against Gram-positive (*S. aureus*, *E. faecalis*, *E. faecium*) and Gram-negative (*K. pneumoniae*, *Salmonella enterica*, *E. coli*) bacteria. Most of the compounds showed weak activity against the tested bacterial strains except for **156**–**158** and **159**, which showed moderate activity against *S. aureus* with MIC values of 64, 32, 16, and 64 µg/mL (a positive control, ampicillin showed MIC = 0.13 µg/mL) whereas **157** and **15**8 showed moderate activity against *S. enterica* with MIC values of 64 µg/mL (ampicillin showed MIC = 0.25 µg/mL). Since sortase A (Srt A), a bacterial cell membrane transpeptidase enzyme that anchors crucial virulence factors to the cell wall surface of Gram-positive bacteria, plays crucial roles in the pathogenesis of Gram-positive but is not necessary for bacterial growth and viability, **155**–**159** were also assayed for their Srt A inhibitory activity using a recombinant SrtA derived from *S. aureus* ATCC6538p. Interestingly, only **156**, **157**, **159**, and **160** showed inhibition of Srt A with IC_50_ values of 62.7, 43.9, 79.4, and 52.4 µM (the positive controls, triphasiol and berberine chloride, showed IC_50_ values of 37.9 and 87.4 µM, respectively) [59].

The bis-indole dihydrospongotine C (**143**) (Figure 16) and the tris-indole tulongicin A (**161**) (Figure 17) were evaluated for their antibacterial activity against Gram-positive *S. aureus* ATCC 29213 and Gram-negative *E. coli* ATCC 25922. Interestingly, both compounds showed strong antibacterial activity against *S. aureus* with MIC values of 3.7 and 1.2 µg/mL, respectively (MIC of a positive control, oxacillin, was 0.25 µg/mL). On the other hand, **143** showed weak activity ((MIC = 100 µg/mL) while **161** was inactive (MIC >100 µg/mL) against *E. coli* at the highest concentration tested (100 μg/mL) [54].

5-Bromotrisindoline (**162**) and 6-bromotrisindoline (**163**) (Figure 17) exhibited moderate to strong antibacterial activity against Gram-positive bacteria, *S. aureus* and *B. subtilis*, with MIC values of 8 and 4 µg/mL, and 16 and 4 µg/mL, respectively. In contrast, both compounds did not display any antibacterial activity against Gram-negative bacteria, *E. coli* and *P. aeruginosa* (MIC ≥ 256 µg/mL). Curiously, the previously described tris-indoline, a non-brominated analog of **162** and **163**, displayed antibacterial activity against *E. coli*, *P. aeruginosa,* and *B. subtilis* [67], implying that introducing a bromine atom into the oxindole moiety of tris-indoline decreases the antibacterial spectrum of these molecules. Interestingly, although **162** and **163** did not exhibit antibacterial activity against *P. aeruginosa,* both compounds, at ½ MIC concentration, were able to inhibit biofilm formation in *P. aeruginosa* PA01 with 49.32% and 41.76% inhibition, respectively (a positive control, azithromycin, showed 52.62% inhibition). Therefore, **162** and **163** could be considered as potential antibiotic adjuvants [60].

The bis-indole alkaloid myrindole A (**164**) (Figure 17) was found to inhibit the growth of *E. coli* and *B. subtilis* with MIC values of 37.5 and 18.5 μM, respectively [61]. The structure of **164** was quite similar to that of dragmacidin E, except for the substitution pattern of the pyrazine ring [67].

The brominated indoles **182**–**185** (Figure 19) from marine alga *Laurencia similis* were evaluated for antibacterial activity against three Gram-positive bacteria (*S. aureus*, *B. subtilis*, *B. thuringensis*) and four Gram-negative (*Pseudomonas lachrymans*, *Agrobacterium tumefaciens*, *Xanthomonas vesicatoria*, and *Ralstonia solanacearum*) bacteria. While **182** exhibited potent inhibitory activity against both Gram-positive and Gram-negative bacterial strains tested with MIC values ranging from 2–8 µg/mL, **183** showed weak activity against both Gram-positive and Gram-negative with MIC values ranging from 12.5–50 µg/mL. Compounds **184** and **185** did not have any effect on the bacterial strains tested (MIC >250 µg/mL), except for **184**, which showed weak activity (MIC = 50 µg/mL against *X. vesicatoria* [65].

### 3.2. Antiviral Activity

Fusariumindole C (**1**), (±)-isoalternatine A (**2**) (Figure 3), fusaindoterpenes A (**48**) and B (**49**) (Figure 6), **and** fusariumindoles A (**75)** and B (**74**) (Figure 10), were evaluated for their antiviral activity against the Zika virus (ZIKV) by a plaque assay on A549 (human lung carcinoma) cell cultures. Compounds **48**, **49**, **74**, and **75** exhibited anti-ZIKV activity with EC_50_ values of 12, 7.5, 50, and 48 μM, respectively. Interestingly, **49** was two-fold more potent against ZIKV than the adenosine analog NITD008, which is a potent inhibitor of ZIKV (NITD008, a positive control, showed an EC_50_ value of 15 μM). Preliminary structure–activity relationship study of these compounds, in comparison with the related compounds previously reported, revealed that the intact pyrrole ring of the indole moiety fused with the cyclopentane ring is required for the activity since introducing an oxygen atom to the pyrrole ring caused a loss of activity in **48** when compared to **49** [7].

The fungal indoleterpenoids, ascandanines B–D (**64**–**66**) (Figure 9), were evaluated for their antiviral effects against the influenza A virus (H1N1) by the cytopathic effect (CPE) inhibition assay. However, only **65** displayed an anti-influenza A effect with IC_50_ = 26 μM, which is more potent than the positive control, ribavirin (IC_50_ = 31 μM) while **64** and **66** were inactive (IC_50_ > 150 μM) [27].

The fungal diketopiperazine indole alkaloid, raistrickindole A (**90**) (Figure 11), was assayed for its in vitro inhibitory activity against the hepatitis C virus (HCV) life cycles. Compound **90** showed an anti-HCV activity with an EC_50_ value of 5.7 μM, which is many folds higher than the EC_50_ value (0.05 μM) of the positive control, VX-950 [39]. Scequinadolines A (**106**), D (**109**), E (**110**) (Figure 12), G (**7**) (Figure 3), and scedapins A (**112**), C **(114**) (Figure 12), and E (**94**) (Figure 11) were also tested for activity against HCV. However, only **106** and **114** displayed significant anti-HCV activity against the J8CC recombinant with EC_50_ values of 128.60 and 110.35 µM, respectively [12].

Dihydrospongotine C (**143**) (Figure 16) and tulongicin A (**161**) (Figure 17) were evaluated for their anti-HIV activity in HIV infectivity assays against the CCR5-tropic primary isolate YU2 and the CXCR4-tropic strain HxB2. Compounds **143** and **161** displayed IC_50_ values of 3.5 and 3.9 µM against YU2 and 4.5 and 2.5 µM against HxB2, respectively [54].

### 3.3. Anticancer Activity

17-*O*-Ethylnotoamide M (**16**), 17-hydroxynotoamide D (**23**) and 10-*O*-ethylnotoamide R (**33**) (Figure 4) were evaluated for their effects on the viability of human non-malignant and prostate cancer cells as well as on the colony formation of human prostate cancer cells 22Rv1, which are known to be resistant to hormone therapy including the novel second generation drugs abiraterone and enzalutamide due to the presence of androgen receptor splice variant AR-V7. Compound **16** did not display cytotoxicity to non-malignant human lung (MRC-9) and human kidney (HEK 293 T) cell lines as well as malignant prostate cancer (22Rv1, PC-3, and LNCaP) cell lines at concentrations up to 100 µM after 48 h of treatment. Moreover, **16** inhibited the colony formation of 22Rv1 cells at a concentration of 100 µM and significantly decreased colony formation at a concentration of 10 µM by 25% [17].

Ascandinines A–D (**63**–**66**) (Figure 9) were also evaluated for their cytotoxicity against MGC-803 (human gastric carcinoma), HCT-116 (human colorectal), SH-SY5Y (human neuroblastoma), Hela, and HL-60 (promyelocytic leukemia) cell lines. However, only **66** was active against the HL-60 cells with an IC_50_ value of 7.8 μM (the IC_50_ value of a positive control, adriamycin, was 0.02 μM) [27].

Asperindoles A (**67**) and C (**69**) (Figure 9) were assayed for cell viability, cell cycle progression, and induction of apoptosis in human prostate cancer cell lines 22Rv1, PC-3, and LNCaP. Compound **67** exhibited cytotoxicity in all three cell lines, with IC_50_ values of 69.4, 47.8, and 4.86 µM, respectively (a positive control, docetaxel, showed IC_50_ values of 15.4, 3.8, and 12.7 nM, respectively). Compound **67** was able to induce apoptosis in 22Rv1 cells at low-micromolar concentration and revealed the S-phase arrest in cell cycle progression analysis. In contrast, **69** was non-cytotoxic against PC-3, LNCaP (androgen-sensitive human prostrate adenocarcinoma cells), and 22Rv1 cell line with IC_50_ >100 µM. Moreover, 22Rv cells treated with 100 µM of **69** displayed only minimal induction of apoptosis as well as no significant changes in cell cycle progression [28].

A fungal *bis*-indolylbenzenoid, candidusin D (**80**) (Figure 10), was tested for its cytotoxic effects against eight cancer cell lines, *viz*. Hep G2 (human hepatocellular carcinoma), HT29 (human colorectal adenocarcinoma), HCT116 (human colorectal carcinoma), A549, A375 (human malignant melanoma), MCF7 (human mammary gland adenocarcinoma), U251 (human glioblastoma multiforme), and T98G (human glioblastoma astrocytoma) by the MTT assay. At 100 µM concentration, **80** exhibited a significant decrease in cell viability in all cell lines tested except for T98G and HepG2. Compound **80** displayed IC_50_ values of 118.9, 111.6, 73.2, 105.8, 123.1, 186.8, and 212.5 µM toward the Hep G2, HT29, HCT116, A549, A375, MCF7, and U251 cell lines, respectively. Doxorubicin, a positive control, showed IC_50_ values of 0.12, 0.63, 0.29, 0.24, 0.05, 0.36, and 1.11 µM, respectively [33].

Penochalasins I (**81**), J (**82**) and K (**83**) (Figure 10) were evaluated for their cytotoxicity against human breast cancer cell line (MDA-MB-435), human gastric cancer cell line (SGC-7901), and A549 by the MTT (3-(4,5-dimethylthiazol-2-yl)-2,5-diphenyl-2H-tetrazolium bromide) method. Compounds **81**–**83** exhibited a broad-spectrum inhibitory activity against all three cell lines. Compound **83** exhibited the strongest activity, with IC_50_ values of 4.65, 5.52, and 8.73 µM toward MDA-MB-435, SGC-7901, and A549, respectively, followed by **81** with IC_50_ values of 7.55, 7.32, and 16.13 µM, respectively. Compound **82** displayed the weakest activity with IC_50_ values of 36.68, 37.70, and 35.93 µM, respectively (IC_50_ values of epirubicin, a positive control, were 0.56, 0.37, and 0.61 µM, respectively) [34,35].

The chlorinated bis-indole alkaloids, dienomycin (**119**) and 6-methoxy-7′,7″-dichlorochromopyrrolic acid (**120**) (Figure 13), were assayed for their cytotoxicity against MDA-MB-435, MDA-MB-231 (human breast adenocarcinoma), NCI-H460 (human non-small-cell lung cancer), HCT-116, HepG2 cancer cell lines, and non-cancerous MCF10A (human breast epithelial) cells. Compound **119** showed stronger activity (IC_50_ values of 3.9, 11.2, 3.6, 4.3, and 8.2 µM, respectively) than **120** (IC_50_ values of 19.4, >25, >25, 13.1, and 18.9 µM, respectively) against all the cancer cell lines but comparable activities against non-cancerous MCF10A cells (IC_50_ values of 3.1 and 2.9 µM) (epirubicin was used as a positive control) [46].

The bromide salt of a synthetic version of tricepyridinium (**122**) (Figure 13) displayed cytotoxicity against murine leukemia P388 cells with an IC_50_ value of 1.0 μM. Comparison of the cytotoxicity among various synthetic analogs revealed that the presence of the indole group at the ethyl side chain of the pyridinium ring of **122** did not enhance cytotoxicity against P388 cells. This structure−activity relationship assessment indicated that the two indole moieties played a concerted role with the 1-ethylpyridinium moiety to induce cytotoxicity of P388 cells. [47].

The sponge-derived (+)-oxoaplysinopsin C (**133a**) and (−)-oxoaplysinopsin C (**133b**) (Figure 14) were tested for their cytotoxicity against the human cervical cancer (HeLa) cell line. Interestingly, **133a** showed two-fold stronger activity (IC_50_ = 27.0 µM) than **133b** (IC_50_ = 61.6 µM). Doxorubicin (IC_50_ = 0.442 µM) was used as a positive control [50].

Dragmacidins G (**141**) and H (**142**) (Figure 16), isolated from a marine sponge *Lipastrotethya* sp., showed toxicity against HeLa cells with IC_50_ values of 4.2 µM and 4.6 µM, respectively [53]. Later on, **142**, isolated from a deep-water marine sponge of an unidentified species of *Spongosorites*, was assayed against a panel of pancreatic cancer cell lines and exhibited a moderate cytotoxicity against PANC-1 (human pancreatic carcinoma), MIA PaCa-2 (human pancreatic carcinoma), BxPC-3 (human pancreatic adenocarcinoma), and ASPC-1 (human pancreatic adenocarcinoma) with IC_50_ values of 18 µM, 26 µM, 14 µM, and 27 µM, respectively [52]. Dragmacidins I (**153**) and J (**154**) (Figure 17) were assayed for their antiproliferative activity toward three human cancer cell lines: A549, HT29, and MDA-MB-231. Compounds **153** and **154** showed significant cytotoxicity against all of the tested cell lines. Compound **153** showed higher potency (IC_50_ values of 2.7, 3.3, 4.7 µM) against A549, HT29, and MDA-MB-231 than **154** (IC_50_ values of 4.7, 4.8, 7.7 µM). Moreover, a mitotic arrest in the metaphase in HeLa cells after treatment for 24 h of **153** and **154,** at a concentration of 10 μM, was independent of the disorganization of the tubulin cytoskeleton. Moreover, A549 cells treated with **153** and **154** at a concentration of 10 μM did not show any effect on their cytoskeleton and were not multinucleated. Furthermore, in the treatment with 10 μM of **153** and **154** for 1, 6, and 24 h, a continuous increment of the retinoblastoma protein (pRB) phosphorylation status in HeLa cells was observed, showing that **153** and **154** effectively inhibited PP1 and/or PP2A phosphatases. It is worth mentioning that pRB is an essential cell cycle controller that is regulated through dephosphorylation by PPs, PP1, and PP2A. Similar results were also observed in A549 cells where the hyperphosphorylation of pRB increased after 1 and 6 h of incubation, but subsequently diminished, resulting in a hypophosphorylated protein after 24 h [58].

Spongosoritins A–D (**155**–**158**) (Figure 17) displayed weak cytotoxicity against A549 and K562 (human immortalized myelogenous leukemia) cancer cell lines with IC_50_ values ranging from 24.2 to 77.3 µM (IC_50_ values of doxorubicin, a positive control, were 0.64 and 0.92 µM, respectively [59].

Securamines H–J (**179**–**181**) (Figure 18) were evaluated for the in vitro cytotoxicity against A2058 (melanoma), HT-29, and MCF-7 (breast adenocarcinoma) cancer cell lines as well as one non-malignant cell line (MRC-5, lung fibroblasts). Compounds **179** (IC_50_ values of 1.4, 1.9, 2.1, 2.7 µM) and **180** (IC_50_ values of 2.7, 2.5, 2.4, 5.3 µM) showed potent cytotoxicity against all the tested cell lines whereas **181** displayed no significant activity at the highest concentration tested (50 μM). The authors concluded that a double bond between C-2 and C-3 is crucial for the cytotoxicity. Compound **179** was evaluated for the cell death kinetics by treating A2058, HT-29, MCF-7, and MRC-5 cells with different concentrations of **179** for 4, 24, 48, and 72 h. Since the IC_50_ values decreased with increasing exposure times, it was concluded that the cytotoxic activity of **179** was time-dependent [64].

### 3.4. Anti-Inflammatory Activity

Asperversamides F–H (**17**–**19**) (Figure 4) and asperversamides A–E (**34**–**38**) (Figure 6) were assayed for their inhibitory effects on inducible nitric oxide synthase (iNOS) and nitic oxide (NO) production. However, only **17**, **18**, **35**, and **36** exhibited potential iNOS inhibitory activities with IC_50_ values of 13.86, 5.39, 9.95, and 16.58 µM, respectively (the IC_50_ value of a positive control, MG132, was 0.24 µM) and inhibited the release of NO in lipopolysaccharide (LPS)-induced Raw264.7 cells, with IC_50_ values of 23.72, 11.17, 17.24, and 25.09 µM, respectively (the IC_50_ value of a positive control, MG132, was 0.17 µM). Since the tested compounds exhibited weak activity (IC_50_ values more than 100 µM) against Raw264.7 cells, their inhibitory effects were not related to cell viability. Moreover, molecular docking studies between **18** and iNOS revealed that **18** adopted an extended conformation and fit well into the ligand binding site of the mutant iNOS. Moreover, hydrogen bonds were predicted between the carbonyl (C-12) and Asn115 as well as between the carbonyl (C-18) and Gln257, in addition to a possible π−π stacking interaction between the diketopiperazine or the double bond at C-10 and the HEME. The results of molecular docking led the authors to conclude that the planarity of the molecule is important for its binding capacity, as this conformation provides enough space for the two carbonyls to form strong hydrogen bonds with the HEME [18].

Asperthins A (**41**), E (**45**), and F (**46**) (Figure 6) were assayed for their anti-inflammatory activity in the *Propionibacterium acnes*-induced human monocyte cell line (THP-1) by measuring the secretion of inflammatory factor 1L-1β by THP-1 cells using the ELISA method. Compounds **41**, **45,** and **46** displayed moderate inti-inflammatory activity with IC_50_ values of 1.46, 30.5, and 37.2 µM, respectively (a positive control, tretinoin, showed IC_50_ = 3.38 µM). Parallel to the IC_50_ values, the safety concentration ranges of **41**, **45**, and **46**, which caused over 80% of cell viability of THP-1 cells, were 0–5, 0–40, and 0–50 µM, respectively [22].

Sponge-derived diketopiperazine-containing bromooxindoles, geobarrettins A–C (**138**–**140**) (Figure 15), were assayed for their anti-inflammatory activity on human dendritic cell (DC) secretion of pro-inflammatory cytokine IL-12p40 and anti-inflammatory cytokine IL-10 production. At a concentration of 10 µg/mL, during 24 h, **139** decreased DC secretion of IL-12p40 by 29% without affecting the secretion of IL-10 while **140** slightly decreased the DC secretion of IL-12p40 by 13% but increased IL-10 production by 40%. Moreover, it was found that the effects of **139** and **140** on IL-12p40 and IL-10 secretion by DCs were not dose-dependent, although **139** showed a tendency of an increasing effect on the secretion of IL-12p40 at higher concentrations. To further elucidate the anti-inflammatory activity of the compounds, DCs matured in the presence of **139** and **140** were co-cultured with allogeneic CD4^+^ T cells and the differentiation of the T cells was investigated by determination of the secretion of the cytokines IL-10, IL-17, and IFN-γ. It was found that T cells co-cultured with DCs matured in the presence of **139** and **140** secreted less IL-10 and IFN-γ than T cells co-cultured with DCs matured without the compounds, however, maturing DCs in the presence of **139** and **140** did not affect T cell secretion of IL-17 [51].

The prenylated indoles, flustramines Q−W (**166**–**172**) and flustraminols C−H (**173**–**178**) (Figure 18), were evaluated for their anti-inflammatory activities by evaluating their effects, at a concentration of 10 μg/mL, on DC secretion of the cytokines IL-12p40 and IL-10 and comparing them to the effect of solvent alone. This model is based on the fact that if a compound either decreases DC secretion of IL-12 or increases secretion of IL-10, it is considered to have an anti-inflammatory effect. The results showed that **166**, **168**, **170**, and **178** decreased DC secretion of the pro-inflammatory cytokine IL-12p40 in the range of 32% to 55% whereas **169** increased DC secretion of the anti-inflammatory cytokine IL-10 by 19−30%, and therefore had modest anti-inflammatory effects. Since none of the active compounds affected the viability of DCs at 10 μg/mL, it was concluded that the decrease in IL12p40 secretion was not due to impaired cell viability [63].

### 3.5. Antidiabetic Activity

Scequinadoline J (**6**) (Figure 3) was screened for its potentiality as an anti-diabetes mellitus type 2 (T2DM) agent by using the 3T3-L1 fibroblast cell line. Compound **6** was found to promote triglyceride (TG) accumulation in 3T3-L1 cells with an EC_50_ value of 1.03 µM (rosiglitazone, a clinically used medication for T2DM, was used as a positive control and showed an EC_50_ value of 0.12 µM). Moreover, **6** did not show cytotoxicity against 3T3-L1 cells at a concentration of 50 µM. Furthermore, it was found that **6** promoted TG accumulation mainly by facilitating adipogenesis and not lipogenesis, which is similar to rosiglitazone [11].

The enzymes protein tyrosine phosphatases (PTPs), which catalyze the dephosphorylation of phosphorylated protein molecules, are essential regulators of signal transduction pathways, which are associated with diabetes, cancer, autoimmune disease, and other diseases. Therefore, they are thought to be promising targets for drug discovery, especially for diabetes. For these reasons, penerpenes A–I (**50**–**58**) (Figure 7) were assayed for their inhibitory activities against PTPs including non-transmembrane PTPs (PTP1B and TCPTP), receptor-like PTP (PTPsigma), and dual-specificity phosphatases (VHR). Compounds **50** (IC_50_ = 1.7 and 5.0 µM against PTP1B and TCPTP, respectively) and **51** (IC_50_ = 2.4 and 4.5 µM, respectively) displayed inhibitory activities with potencies comparable to that of Na3VO4 (IC_50_ = 1.6 and 2.4 µM, respectively) while **54** (IC_50_ = 14 µM against PTP1B)**, 55** (IC_50_ = 27 µM against PTP1B), and **57** (IC_50_ = 23 and 25 µM against PTP1B and TCPTP) were ca. ten-fold less active than Na3VO4 (IC_50_ = 2 and 2 µM, respectively). On the other hand, **50** (IC_50_ = 29 and >30 µM, respectively) and **51** (IC_50_ = > 30 µM) exhibited weak activity against PTPsigma and VHR when compared to Na3VO4 (IC_50_ = 9.7 and 13.2 µM, respectively) while **57** was only weakly active against PTPsigma with IC_50_ = 38 µM (IC_50_ of Na3VO4 = 10 µM) [24,25]. Docking study suggested that **50** binds deep in the active site pocket and forms hydrogen bonds with Asp 181 and Gln 262. However, with a larger ring structure, **51** did not bind at the active site but instead interacted with Phe 30 in the so-called secondary binding site of PTP1B [25]. SF5280-415 (**116**) (Figure 12) displayed an inhibitory effect against PTP1B, a promising therapeutic target for the treatment of diabetes and obesity, with an IC_50_ value of 14.2 μM [43].

Since hypoadiponectinemia is a major symptom of diverse human metabolic diseases such as obesity, type 2 diabetes, atherosclerosis, and non-alcoholic steatohepatitis, compounds that stimulate adiponectin secretion could be promising for the treatment of metabolic diseases such as T2DM. For these reasons, psammocindoles A–C (**123**–**125**) (Figure 14) were evaluated for the adiponectin-secretion-stimulating activities in hBM-MSCs using an adipogenic cocktail consisting of insulin, dexamethasone, and isobutyl methylxanthine (IDX). Compounds **123**–**125** (at 10 μM) significantly increased adiponectin secretion during adipogenesis in hBM-MSCs compared to that in the IDX control. In a concentration-response analysis, **123** and **124** were found to be more potent (EC_50_ = 9.86 and 6.20 μM, respectively) than bezafibrate (EC_50_ > 10 μM), a currently prescribed pan-PPAR agonist, while **125** was less potent (EC_50_ > 10 μM). Moreover, **123** and **124** also increased the lipid accumulation in differentiated adipocytes in hBM-MSCs compared to that of the IDX control, suggesting that, similar to pioglitazone, **123** and **124** are able to improve insulin sensitivity. Thus, these compounds could represent a novel pharmacophore for the development of therapeutic agents for the treatment of human metabolic diseases [48]. (±)-Oxoaplysinopsin B (**132a/132b**) (Figure 14) showed inhibitory activity against PTP1B with an IC_50_ value of 20.8 µM. Interestingly, the optically pure (+)-**132a** was more potent (IC_50_ = 18.3 µM) than both the racemate and (−)-**132a** (IC_50_ = 26.5 µM) [50].

### 3.6. Antiparasitic Activity

Dragmacidin G (**141**) (Figure 16) was assayed against a chloroquine-resistant (DD2) *Plasmodium falciparum* strain and displayed a modest inhibitory activity with IC_50_ = 6.4 µM. The selectivity determination using the MTS [(3-(4,5-dimethylthiazol-2-yl)-5-(3-carboxymethoxyphenyl)-2-(4-sulfophenyl)-2H-tetrazolium)] assay showed that **141** was almost equally cytotoxic to the NIH 3T3 mouse fibroblast cell line (IC_50_ = 7.8 µM), indicating no selectivity for the malaria parasite [52].

5-Bromotrisindoline (**162**) and 6-bromotrisi3ndoline (**163**) (Figure 17) were evaluated for their antitrypanosomal activity against *Trypanosoma brucei* that causes African trypanosomiasis, also known as sleeping sickness. Compounds **162** and **163** demonstrated moderate antitrypanosomal activity after 48 and 72 h, with IC_50_ values of 15.36 (48 h) and 13.47 (72 h), and 12.35 (48 h), and 10.27 (72 h) µM, respectively [60],

### 3.7. Neuroprotective Activity

The deoxyisoaustamide derivatives **24**–**30** (Figure 5) were examined for their potential protective effects against the acute toxicity of paraquat (PQ) in murine neuroblastoma Neuro-2a cells. Treatment of Neuro-2a cells with 500 µM of PQ induced a decrease in cell viability by 51.8%. However, co-treatment with 1 µM of **27** and **29** increased a viability of PQ-treated cells by 38.6% and 30.3%, respectively, whereas **28** increased a viability of the cells by 36.5% and 39.4% at concentrations of 1 µM and 10 µM, respectively. Moreover, **27, 28**, and **29** were not cytotoxic to Neuro-2a cells [17].

### 3.8. Enzyme Inhibitors

Guitarrins A–E (1**26**–**130**) (Figure 14) were assayed for the inhibitory activity against the enzyme alkaline phosphatase (ALP) from the marine bacterium *Cobetia marina.* Reasons for interest in this ALP are its possible application as a tool for structural and functional studies of nucleic acids as well as for the preparation of Ig-enzyme conjugates for immunologic assays. ALP produced by *C. marina* was found to have very high specific activity. Compound **128** was found to be a potent inhibitor of ALP from *C. marina* with an IC_50_ value of 2.0 μM when compared to a positive control, ethylenediaminetetraacetic acid (EDTA, IC_50_ = 80,000 μM), which is the most active inhibitor of *C. marina* alkaline phosphatase (CmAP) described to date. On the other hand, **129** showed weak inhibitory activity (IC_50_ = 100 μM) whereas **126, 127**, and **130** did not inhibit this enzyme [49].

### 3.9. Other Activities

Dinotoamide J (**73**) (Figure 9) was tested for its pro-angiogenic activity in a vatalanib (PTK787)-induced vascular injury zebrafish model. At a concentration of 70 µg/mL, **73** exhibited a significant effect in a dose-dependent manner. Ginsenoside Rg1 (120 µg/mL) was used as a positive control [30].

In order to facilitate the localization of the bioactive indole alkaloids from different sources and their biological and pharmacological activities, the source organisms, biological/pharmacological activities, compound numbers, and the references cited are summarized in Table 1.

## 4. Conclusions

Marine organisms have been proven to be valuable sources of structurally diverse and unique indole compounds. Their structures vary from simple to complex and incorporate different types of compounds ranging from simple prenyl groups to diterpene moieties or other molecules or scaffolds such as pyrazine, diketopiperazine, guanidine, and quinazoline. The indole ring system can be present as an intact indole or undergoes oxidation or/and rearrangements to form different scaffolds or even incorporate a nitrogen atom to form azaindole derivatives or halogen and sulfur atoms to form bromoindoles, sulfonyl, and sulfinyl side chains. Moreover, these marine-derived indoles can have a single indole unit or contain two or three indole units as found in bis-indoles, tris-indoles, homo-, and heterodimers of indole compounds. This structural diversity of marine-derived indoles is reflected by a variety of biological and pharmacological activities that they manifest such as antibacterial, antiviral, anticancer, antiparasitic activities, and enzyme inhibition. Thus, marine-derived indole compounds are excellent sources to be explored for pharmacophores to serve for the development of drug leads or even therapeutic agents for the treatments of infectious diseases, cancer, or even some metabolic diseases.

## Figures and Tables

**Table 1:** Summary of source organisms, biological/pharmacological activities, compound numbers, and the cited references.

Organisms	Biological/Pharmacological Activities	Compound	Reference
**1. Marine-derived fungi**			
**Antibacterial activity**			
*Aspergillus* sp. YJ191021	*Xanthomonas oryzae pv. oryzae* *X. oryzae pv. Oryzicola* *Edwardsiella tarda* *Vibrio anguillarum* *V. parahaemolyticus* *Aeromonas hydrophilia*	**41**	[22]
*A. fumigatus* SCSIO 41012	*Acinetoobacter baumannii* ATCC 19606, *A. baumannii* ATCC 15122, *Klebsiella pneumoniae* ATCC 14578 *Staphylococcus aureus* ATCC 16339	**93**	[41]
**Antibiofilm activity**			
*Eurotium chevalieri* KUFA 0006	*S. aureus* ATCC 25923	**9**	[14]
**Anti-Quorum sensing activity**			
*Aspergillus* sp. HNMF114	*Chromobacterium violaceum* CV026	**91**, **92**	[40]
**Antifungal activity**			
*Aspergillus* sp. YJ191021	*Rhizoctonia solani*	**45**	[22]
*A. fumigatus* SCSIO 41012	*Fusarium oxysporum* f. sp. *cucumerinum* *F. oxysporum* f. sp. *momordicae*	**93**	[41]
*Penicillium chrysogenum* V11	*Colletotrichum gloeosporioides* *R. solani*	**82**, **83**	[34,35]
**Antiviral activity**			
*Fusarium* sp. L1	Anti-Zika virus	**48**, **49**, **74**, **75**	[7]
*A. candidus* HDN15-152	Anti-influenza A virus (H1N1)	**65**	[27]
*P. raistrickii* IMB17-034	Anti-hepatitis C virus	**90**	[39]
*Scedosporium apiospermum* F41	Anti-hepatitis C virus	**106**, **114**	[12]
**Anticancer activity**			
*Aspergillus* sp. KMM4676	22Rv1, PC-3, and LNCaP	**66**, **67**	[28]
*A. candidus* KUFA0062	Hep G2, HT29, HCT116, A549, A375, MCF7 and U251	**80**	[33]
*P. chrysogenum* V11	MDA-MB-435, SGC-7901,A549	**81**, **83**	[34,35]
**Anti-inflammatory activity**			
*Aspergillus versicolor*	iNOS inhibitory activity	**17**, **18**, **35**, **36**,	[18]
*Aspergillus* sp. YJ191021	Inhibit secretion of 1L-1β by THP-1 cells	**41**, **45**, **46**	[22]
**Antidiabetic activity**			
*S. apiospermum* F41-1	promote triglyceride accumulation in 3T3-L1	**6**	[12]
*Penicillium* sp. KFD28	Inhibition of protein tyrosine	**50**, **51**, **54**,	[24]
*Aspergillus* sp. SF-5280	phosphatases (PTPs)	**55**, **57**	[25]
	Inhibition of non-transmembrane PTPs (PTP1B)	**116**	[43]
**Neuroprotective activity**			
*P. dimorphosporum* KMM 4689	increased a viability of paraquat-treated cells	**27**, **29**	[30]
**Pro-angiogenic activity**			
*A. austroafricanus* Y32-2	Pro-angiogenic activity in a vatalanib (PTK787)-induced vascular injury zebrafish model	**73**	[30]
**2.** **Marine-derived bacteria**			
**Antibacterial activity**			
*Streptomyces* sp. SCSIO 11791	*Micrococcus luteus* ML01, *S. aureus* ATCC 29213, and a panel of MRSA isolated from human patients (MRSA 991, MRSA 1862, MRSA 669 A, MRSA A2) and pig (MRSA GDQ6P012P, MRSA GDE4P037P)	**119**, **120**	[46]
*Acinetobacter* sp. ZZ1275	*S. aureus* (MRSA), *E. coli*	**118**, **121**	[45]
*E. coli* transfected by metagenomic DNA prepared from the marine sponge *Dicderma calyx*	*Bacillus cereus, S. aureus* (MSSA)	**122**	[47]
**Antifungal activity**			
*Acinetobacter* sp. ZZ1275	*Candida albicans*	**118**, **121**	[45]
**Anticancer activity**			
*Streptomyces* sp. SCSIO 11791	MDA-MB-435, MDA-MB-231 NCI-H460, HCT-116, HepG2. MCF10A	**119**, **120**	[46]
**3.** **Marine sponges**			
**Antibacterial activity**			
*Spongosorites* sp.	*Mycobacterium tuberculosis* CDC1551	**141**	[52]
*Spongosorites* sp.	*S. aureus*	**156**–**158**, **159**	[59]
	*S. entérica*	**158**, **159**	[59]
*Topsentia* sp.	*S. aureus* ATCC 29213	**143**, **161**	[54]
*Callyspongia siphonella*	*S. aureus* and *B. subtilis*	**162**, **163**,	[60]
*Myrmekioderma* sp.	*E. coli* and *B. subtilis*	**164**	[67]
**Antiviral activity**			
*Topsentia* sp.	Anti-HIV activity	**143**, **161**	[54]
**Anticancer activity**			
*Fascaplysinopsis reticulata*	HeLa	**133a**, **133b**	[50]
*Spongosorites* sp.	Human pancreatic cell lines: PANC-1, MIA PaCa-2, BxPC-3, ASPC-1	**141**	[52]
*Dragmacidon* sp.	A549, HT29, and MDA-MB-231	**153**, **154**	[58]
*Spongosorites* sp.	A549 and K562	**155**–**158**	[59]
**Anti-inflammatory activity**			
*Geodia barretti*	Decrease dendritic cell secretion of pro-inflammatory cytokine IL-12p40 and anti-inflammatory cytokine IL-10 production	**139**, **140**	[59]
**Antidiabetic activity**			
*Psammocinia vermis*	Increase adiponectin secretion during adipogenesis in hBM-MSCs	**123**–**125**	[48]
*Fascaplysinopsis reticulata*	inhibitory activity against PTP1B	**132a**, **132b**	[50]
**Antiparasitic activity**			
*Callyspongia siphonella*	Antitrypanosomal activity against *Trypanosoma brucei*	**162**, **163**	[60]
**Enzyme inhibitors**			
*Guitarra fimbriata*	Inhibitor of alkaline phosphatase	**128**	[49]
*Spongosorites* sp.	Inhibit sortase A	**156**, **157**, **159**, **160**	[59]
**4.** **Bryozoans**			
**Anticancer activity**			
*Securiflustra securifrons*	A2058, HT-29, MCF-7, MRC-5	**179**, **180**	[64]
**Anti-inflammatory activity**			
*Flustra foliácea*	Decrease DC secretion of the pro-inflammatory cytokine IL-12p40	**166**, **168**, **170**, **178**	[63]
**5.** **Algae**			
**Antibacterial activity**			
*Laurencia similis*	*S. aureus, B. subtilis, B. thuringensis*, *Pseudomonas lachrymans*, *Agrobacterium tumefaciens*, *Xanthomonas vesicatória*, *Ralstonia solanacearum*	**182**, **183**	[65]

## References

[1] Harvey A.L., Edrada-Ebel R., Quinn R.J. (2015). The re-emergence of natural products for drug discovery in the genomics era. Nat. Rev. Drug Discov..

[2] Newman D.J., Cragg G.M. (2016). Drugs and drug candidates from marine sources: An assessment of the current “state of play”. Planta Med..

[3] Gul W., Hamann M.T. (2005). Indole alkaloid marine natural products: An established source of cancer drug leads with considerable promise for the control of parasitic, neurological and other diseases. Life Sci..

[4] França P.H., Barbosa D.P., da Silva D.L., Ribeiro Ê.A., Santana A.E., Santos B.V., Barbosa-Filho J.M., Quintans J.S., Barreto R.S., Quintans-Júnior L.J. (2014). Indole alkaloids from marine sources as potential leads against infectious diseases. BioMed Res. Int..

[5] Netz N., Opatz T. (2015). Marine indole alkaloids. Mar. Drugs.

[6] Kochanowska-Karamyan A.J., Hamann M.T. (2010). Marine indole alkaloids: Potential new drug leads for the control of depression and anxiety. Chem. Rev..

[7] Guo Y.W., Liu X.J., Yuan J., Li H.J., Mahmud T., Hong M.J., Yu J.C., Lan W.J. (2020). l-Tryptophan induces a marine-derived *Fusarium* sp. to produce indole alkaloids with activity against the Zika virus. J. Nat. Prod..

[8] Chen Y.X., Xu M.Y., Li H.J., Zeng K.J., Ma W.Z., Tian G.B., Xu J., Yang D.P., Lan W.J. (2017). Diverse secondary metabolites from the marine-derived fungus *Dichotomomyces cejpii* F31-1. Mar. Drugs.

[9] Wang K.T., Xu M.Y., Liu W., Li H.J., Xu J., Yang D.P., Lan W.J., Wang L.Y. (2016). Two additional new compounds from the marine-derived fungus *Pseudallescheria ellipsoidea* F42-3. Molecules.

[10] Meng L.H., Chen H.Q., Form I., Konuklugil B., Proksch P., Wang B.G. (2016). New chromone, isocoumarin, and indole alkaloid derivatives from three sponge-derived fungal strains. Nat. Prod. Commun..

[11] Li C.J., Chen P.N., Li H.J., Mahmud T., Wu D.L., Xu J., Lan W.J. (2020). Potential antidiabetic fumiquinazoline alkaloids from the marine-derived fungus *Scedosporium apiospermum* F41-1. J. Nat. Prod..

[12] Huang L.H., Xu M.Y., Li H.J., Li J.Q., Chen Y.X., Ma W.Z., Li Y.P., Xu J., Yang D.P., Lan W.J. (2017). Amino acid-directed strategy for inducing the marine-derived fungus *Scedosporium apiospermum* F41–1 to maximize alkaloid diversity. Org. Lett..

[13] May Zin W.W., Buttachon S., Dethoup T., Fernandes C., Cravo S., Pinto M.M.M., Gales L., Pereira J.A., Silva A.M.S., Sekeroglu N. (2016). New cyclotetrapeptides and a new diketopiperzine derivative from the marine sponge-associated fungus *Neosartorya glabra* KUFA 0702. Mar. Drugs.

[14] May Zin W.W., Buttachon S., Dethoup T., Pereira J.A., Gales L., Inácio Â., Costa P.M., Lee M., Sekeroglu N., Silva A.M.S. (2017). Antibacterial and antibiofilm activities of the metabolites isolated from the culture of the mangrove-derived endophytic fungus *Eurotium chevalieri* KUFA 0006. Phytochemistry.

[15] Zhong W.M., Wang J.F., Shi X.F., Wei X.Y., Chen Y.C., Zeng Q., Yao Xiang Y., Chen X.Y., Tian X.P., Xiao Z.H. (2018). Eurotiumins A–E, five new alkaloids from the marine-derived fungus *Eurotium* sp. SCSIO F452. Mar. Drugs.

[16] Wei X., Feng C., Wang S.Y., Zhang D.M., Li X.H., Zhang C.X. (2020). New indole diketopiperazine alkaloids from soft coral-associated epiphytic fungus *Aspergillus* sp. EGF 15-0-3. Chem. Biodivers..

[17] Afiyatullov S.H., Zhuravleva O.I., Antonov A.S., Berdyshev D.V., Pivkin M.V., Denisenko V.A., Popov R.S., Gerasimenko A.V., von Amsberg G., Dyshlovoy S.A. (2018). Prenylated indole alkaloids from co-culture of marine-derived fungi *Aspergillus sulphureus* and *Isaria felina*. J. Antibiot..

[18] Li H., Sun W., Deng M., Zhou Q., Wang J., Liu J., Chen C., Qi C., Luo Z., Xue Y. (2018). Asperversiamides, linearly fused prenylated indole alkaloids from the marine-derived fungus *Aspergillus versicolor*. J. Org. Chem..

[19] Lan W.J., Wang K.T., Xu M.Y., Zhang J.J., Lam C.K., Zhong G.H., Xu J., Yang D.P., Li H.J., Wang L.Y. (2016). Secondary metabolites with chemical diversity from the marine-derived fungus *Pseudallescheria boydii* F19-1 and their cytotoxic activity. RSC Adv..

[20] Zhuravleva O.I., Antonov A.S., Trang V.T.D., Pivkin M.V., Khudyakova Y.V., Denisenko V.A., Popov R.S., Kim N.Y., Yurchenko E.A., Gerasimenko A.V. (2021). New deoxyisoaustamide derivatives from the coral-derived fungus *Penicillium dimorphosporum* KMM 4689. Mar. Drugs.

[21] Xu X., Zhang X., Nong X., Wang J., Qi S. (2017). Brevianamides and mycophenolic acid derivatives from the deep-sea-derived fungus *Penicillium brevicompactum* DFFSCS025. Mar. Drugs.

[22] Yang J., Gong L., Guo M., Jiang Y., Ding Y., Wang Z., Xin X., An F. (2021). Bioactive indole diketopiperazine alkaloids from the marine endophytic fungus *Aspergillus* sp. YJ191021. Mar. Drugs.

[23] Zheng Y.Y., Shen N.X., Liang Z.Y., Shen L., Chen M., Wang C.Y. (2020). Paraherquamide J, a new prenylated indole alkaloid from the marine-derived fungus *Penicillium janthinellum* HK1-6. Nat. Prod. Res..

[24] Kong F.D., Fan P., Zhou L.M., Ma Q.Y., Xie Q.Y., Zheng H.Z., Zheng Z.H., Zhang R.S., Yuan J.Z., Dai H.F. (2019). Penerpenes A–D, four indole terpenoids with potent protein tyrosine phosphatase inhibitory activity from the marine-derived fungus *Penicillium* sp. KFD28. Org. Lett..

[25] Zhou L.M., Kong F.D., Fan P., Ma Q.Y., Xie Q.Y., Li J.H., Zheng H.Z., Zheng Z.H., Yuan J.Z., Dai H.F. (2019). Indole-diterpenoids with protein tyrosine phosphatase inhibitory activities from the marine-derived fungus *Penicillium* sp. KFD28. J. Nat. Prod..

[26] Yang B., Tao H., Lin X., Wang J., Liao S., Dong J., Zhou X., Liu Y. (2018). Prenylated indole alkaloids and chromone derivatives from the fungus *Penicillium* sp. SCSIO041218. Tetrahedron.

[27] Zhou G., Sun C., Hou X., Che Q., Zhang G., Gu Q., Liu C., Zhu T., Li D. (2021). Ascandinines A–D, indole diterpenoids from the sponge-derived fungus *Aspergillus candidus* HDN15-152. J. Org. Chem..

[28] Ivanets E.V., Yurchenko A.N., Smetanina O.F., Rasin A.B., Zhuravleva O.I., Pivkin M.V., Popov R.S., Von Amsberg G., Afiyatullov S.H., Dyshlovoy S.A. (2018). Asperindoles A–D and a *p*-terphenyl derivative from the ascidian-derived fungus *Aspergillus* sp. KMM 4676. Mar. Drugs.

[29] Cai R., Jiang H., Xiao Z., Cao W., Yan T., Liu Z., Lin S.E., Long Y., She Z. (2019). (−)-and (+)-Asperginulin A, a pair of indole diketopiperazine alkaloid dimers with a 6/5/4/5/6 pentacyclic skeleton from the mangrove endophytic fungus *Aspergillus* sp. SK-28. Org. Lett..

[30] Li P., Zhang M., Li H., Wang R., Hou H., Li X., Liu K., Chen H. (2021). New prenylated indole homodimeric and pteridine alkaloids from the marine-Derived fungus *Aspergillus austroafricanus* Y32-2. Mar. Drugs.

[31] Shaker S., Fan R.Z., Li H.J., Lan W.J. (2021). A pair of novel bisindole alkaloid enantiomers from marine fungus *Fusarium* sp. XBB-9. Nat. Prod. Res..

[32] Yuan M.X., Qiu Y., Ran Y.Q., Feng G.K., Deng R., Zhu X.F., Lan W.J., Li H.J. (2019). Exploration of indole alkaloids from marine fungus *Pseudallescheria boydii* F44-1 using an amino acid-directed strategy. Mar. Drugs.

[33] Buttachon S., Ramos A.A., Inácio Â., Dethoup T., Gales L., Lee M., Costa P.M., Silva A.M.S., Sekeroglu N., Rocha E. (2018). Bis-indolyl benzenoids, hydroxypyrrolidine derivatives and other constituents from cultures of the marine sponge-associated fungus *Aspergillus candidus* KUFA0062. Mar. Drugs.

[34] Huang S., Chen H., Li W., Zhu X., Ding W., Li C. (2016). Bioactive chaetoglobosins from the mangrove endophytic fungus *Penicillium chrysogenum*. Mar. Drugs.

[35] Zhu X., Zhou D., Liang F., Wu Z., She Z., Li C. (2017). Penochalasin K, a new unusual chaetoglobosin from the mangrove endophytic fungus *Penicillium chrysogenum* V11 and its effective semi-synthesis. Fitoterapia.

[36] Qi J., Jiang L., Zhao P., Chen H., Jia X., Zhao L., Dai H., Hu J., Liu C., Shim S.H. (2020). Chaetoglobosins and azaphilones from *Chaetomium globosum* associated with *Apostichopus japonicus*. Appl. Microbiol. Biot..

[37] Luo X.W., Gao C.H., Lu H.M., Wang J.M., Su Z.Q., Tao H.M., Zhou X.F., Yang B., Liu Y.H. (2020). HPLC-DAD-guided isolation of diversified chaetoglobosins from the coral-associated fungus *Chaetomium globosum* C2F17. Molecules.

[38] Zhang Z., Min X., Huang J., Zhong Y., Wu Y., Li X., Deng Y., Jiang Z., Shao Z., Zhang L. (2016). Cytoglobosins H and I, new antiproliferative cytochalasans from deep-sea-derived fungus *Chaetomium globosum*. Mar. Drugs.

[39] Li J., Hu Y., Hao X., Tan J., Li F., Qiao X., Chen S., Xiao C., Chen M., Peng Z. (2019). Raistrickindole A, an anti-HCV oxazinoindole alkaloid from *Penicillium raistrickii* IMB17-034. J. Nat. Prod..

[40] Kong F.D., Zhang S.L., Zhou S.Q., Ma Q.Y., Xie Q.Y., Chen J.P., Li J.H., Zhou L.M., Yuan J.Z., Hu Z. (2019). Quinazoline-containing indole alkaloids from the marine-derived fungus *Aspergillus* sp. HNMF114. J. Nat. Prod..

[41] Limbadri S., Luo X., Lin X., Liao S., Wang J., Zhou X., Yang B., Liu Y. (2018). Bioactive novel indole alkaloids and steroids from deep sea-derived fungus *Aspergillus fumigatus* SCSIO 41012. Molecules.

[42] Liu S.S., Yang L., Kong F.D., Zhao J.H., Yao L., Yuchi Z.G., Ma Q.Y., Xie Q.Y., Zhou L.M., Guo M.F. (2021). Three new quinazoline-containing indole alkaloids from the marine-derived fungus *Aspergillus* sp. HNMF114. Front. Microbiol..

[43] Cho K.H., Sohn J.H., Oh H. (2018). Isolation and structure determination of a new diketopiperazine dimer from marine-derived fungus *Aspergillus* sp. SF-5280. Nat. Prod. Res..

[44] Xie C.L., Xia J.M., Su R.Q., Li J., Liu Y., Yang X.W., Yang Q. (2018). Bacilsubteramide A, a new indole alkaloid, from the deep-sea-derived *Bacillus subterraneus* 11593. Nat. Prod. Res..

[45] Anjum K., Kaleem S., Yi W., Zheng G., Lian X., Zhang Z. (2019). Novel antimicrobial indolepyrazines A and B from the marine-associated *Acinetobacter* sp. ZZ1275. Mar. Drugs.

[46] Song Y., Yang J., Yu J., Li J., Yuan J., Wong N.K., Ju J. (2020). Chlorinated bis-indole alkaloids from deep-sea derived *Streptomyces* sp. SCSIO 11791 with antibacterial and cytotoxic activities. J. Antibiot..

[47] Okada M., Sugita T., Wong C.P., Wakimoto T., Abe I. (2017). Identification of pyridinium with three indole moieties as an antimicrobial agent. J. Nat. Prod..

[48] Kwon O.S., Ahn S., Jeon J., Park I.G., Won T.H., Sim C.J., Park H., Oh D.C., Oh K.B., Noh M. (2021). Psammocindoles A–C: Isolation, synthesis, and bioactivity of indole-γ-lactams from the sponge *Psammocinia vermis*. Org. Lett..

[49] Guzii A.G., Makarieva T.N., Denisenko V.A., Gerasimenko A.V., Udovenko A.A., Popov R.S., Dmitrenok P.S., Golotin V.A., Fedorov S.N., Grebnev B.B. (2019). Guitarrins A–E and aluminumguitarrin A: 5-azaindoles from the Northwestern Pacific marine sponge *Guitarra fimbriata*. J. Nat. Prod..

[50] Wang Q., Tang X.L., Luo X.C., de Voog N.J., Li P.L., Li G.Q. (2019). Aplysinopsin-type and bromotyrosine-derived alkaloids from the south China sea sponge *Fascaplysinopsis reticulata*. Sci. Rep..

[51] Di X., Rouger C., Hardardottir I., Freysdottir J., Molinski T.F., Tasdemir D., Omarsdottir S. (2018). 6-bromoindole derivatives from the Icelandic marine sponge *Geodia barretti*: Isolation and anti-Inflammatory activity. Mar. Drugs.

[52] Wright A.E., Killday K.B., Chakrabarti D., Guzmán E.A., Harmody D., McCarthy P.J., Pitts T., Pomponi S.A., Reed J.K., Roberts B.F. (2017). Dragmacidin G, a bioactive bis-indole alkaloid from a deep-water sponge of the genus *Spongosorites*. Mar. Drugs.

[53] Hitora Y., Takada K., Ise Y., Okada S., Matsunaga S. (2016). Dragmacidins G and H, bisindole alkaloids tethered by a guanidino ethylthiopyrazine moiety, from a *Lipastrotethya* sp. marine sponge. J. Nat. Prod..

[54] Liu H.B., Lauro G., O’Connor R.D., Lohith K., Kelly M., Colin P., Bifulco G., Bewley C.A. (2017). Tulongicin, an antibacterial tri-indole alkaloid from a deep-water *Topsentia* sp. sponge. J. Nat. Prod..

[55] Wang D., Feng Y., Murtaza M., Wood S., Mellick G., Hooper J.N., Quinn R.J. (2016). A grand challenge: Unbiased phenotypic function of metabolites from *Jaspis splendens* against Parkinson’s disease. J. Nat. Prod..

[56] Ragini K., Piggott A.M., Karuso P. (2019). Bisindole alkaloids from a New Zealand deep-sea marine sponge *Lamellomorpha strongylata*. Mar. Drugs.

[57] Jennings L.K., Khan N., Kaur N., Rodrigues D., Morrow C., Boyd A., Thomas O.P. (2019). Brominated bisindole alkaloids from the Celtic Sea sponge *Spongosorites calcicola*. Molecules.

[58] Cruz P.G., Leal J.F.M., Daranas A.H., Pérez M., Cuevas C. (2018). On the mechanism of action of dragmacidins I and J, two new representatives of a new class of protein phosphatase 1 and 2A inhibitors. ACS Omega.

[59] Park J.S., Cho E., Hwang J.Y., Park S.C., Chung B., Kwon O.S., Sim C.J., Oh D.C., Oh K.B., Shin J. (2021). Bioactive bis (indole) alkaloids from a *Spongosorites* sp. sponge. Mar. Drugs.

[60] El-Hawary S.S., Sayed A.M., Mohammed R., Hassan H.M., Rateb M.E., Amin E., Mohammed T.A., El-Mesery M., Muhsinah A.B., Alsayari A. (2019). Bioactive brominated oxindole alkaloids from the Red Sea sponge *Callyspongia siphonella*. Mar. Drugs.

[61] Moosmann P., Taniguchi T., Furihata K., Utsumi H., Ise Y., Morii Y., Yamawaki N., Takatani T., Arakawa O., Okada S. (2021). Myrindole A, an antimicrobial bis-indole from a marine sponge *Myrmekioderma* sp. Org. Lett..

[62] Kleks G., Holland D.C., Kennedy E.K., Avery V.M., Carroll A.R. (2020). Antiplasmodial alkaloids from the Australian bryozoan *Amathia lamourouxi*. J. Nat. Prod..

[63] Di X., Wang S., Oskarsson J.T., Rouger C., Tasdemir D., Hardardottir I., Freysdottir J., Wang X., Molinski T.F., Omarsdottir S. (2020). Bromotryptamine and imidazole alkaloids with anti-inflammatory activity from the bryozoan *Flustra foliacea*. J. Nat. Prod..

[64] Hansen K.Ø., Isaksson J., Bayer A., Johansen J.A., Andersen J.H., Hansen E. (2017). Securamine derivatives from the Arctic bryozoan *Securiflustra securifrons*. J. Nat. Prod..

[65] Li M.C., Sun W.S., Cheng W., Liu D., Liang H., Zhang Q.Y., Lin W.H. (2016). Four new minor brominated indole related alkaloids with antibacterial activities from *Laurencia similis*. Bioorg. Med. Chem. Lett..

[66] Cai Y.S., Sun J.Z., Tang Q.Q., Fan F., Guo Y.W. (2018). Acanthiline A, a pyrido[1,2-a] indole alkaloid from Chinese mangrove *Acanthus ilicifolius*. J. Asian Nat. Prod. Res..

[67] Kobayashi M., Aoki S., Gato K., Matsunami K., Kurosu M., Kitagawa I. (1994). Marine natural products. XXXIV. Trisindoline, a new antibiotic indole trimer, produced by a bacterium of *Vibrio* sp. separated from the marine sponge *Hyrtios altum*. Chem. Pharm. Bull..

